# Top-down knowledge surpasses selection history in influencing attentional guidance

**DOI:** 10.3758/s13414-022-02648-3

**Published:** 2023-01-24

**Authors:** Markus Grüner, Florian Goller, Ulrich Ansorge

**Affiliations:** 1 Department of Cognition, Emotion, and Methods in Psychology, University of Vienna, Liebiggasse 5, 1010 Vienna, Austria; 2 Austrian Marketing University of Applied Sciences, Vienna, Austria; 3 Cognitive Science Hub, University of Vienna, Vienna, Austria; 4 Research Platform Mediatised Lifeworlds, University of Vienna, Vienna, Austria

**Keywords:** Attentional guidance, Selection history, Top-down attention, Learning

## Abstract

Visual attention is influenced by the characteristics of the stimuli (bottom-up), their task relevance (top-down), and prior experience (e.g., selection history and learning). However, it is largely unclear how learning and selection history interact with top-down attentional guidance. We combined trial-and-error learning with a spatial cueing protocol to test whether previously learned target-defining features continued to capture attention if participants were instructed to search for a new target feature (Experiment 1) or had to learn a new target feature (Experiment 2). It turned out that the previously learned feature quickly stopped capturing attention when the target feature changed (Experiment 1; even before participants learned the new target-defining feature, in Experiment 2). Finally, in Experiment 3, in which participants learned to search for targets defined by two redundant features (color and orientation), we found possible reasons for the dominance of the instructed feature over learning. Participants reported using only the target color for their search. Consequently, only cues with a target color captured attention. The unused target orientation only captured attention in participants aware of both target-defining features (13 out of 23) and only if the orientation was presented in the target color. We conclude that knowledge of target-defining features and their use as search criterion is critical for attentional guidance, while previously learned target features either influence attentional guidance only contingent on such deliberately selected top-down based attentional control settings or may influence visual search but not attentional guidance.

From the myriads of visual information in the environment, only a fraction is behaviorally relevant at each moment. Hence, a crucial requirement for the function of the visual system is the selection of relevant information for prioritized processing. This selection process is called *visual attentio*n and results in improved processing of selected stimuli compared with nonselected ones. Visual attention was described as the result of bottom-up and top-down processes, as well as of prior experience or “selection history” (Awh et al., 2012).

Understanding how these processes interact under different conditions is critical for predicting human behavior and designing optimal conditions to guide human attention successfully in applied settings (e.g., during human–machine interaction). Thus, the current study addressed to what extent learned target-associated features might continue to guide attention despite instructions to search for a different feature (Experiments 1 and 2), and how awareness and usage of target-defining features interact during attentional guidance (Experiment 3).

To start with bottom-up processes, salient (i.e., conspicuous) stimuli can capture attention in a reflexive or stimulus-driven way (Itti & Koch, 2001; Jonides, 1981; Nothdurft, 1993; Theeuwes, 1991, 1992; Weichselbaum & Ansorge, 2018). For example, stimuli showing a strong local feature contrast to their adjacent regions, such as singletons, can capture attention automatically (i.e., without voluntary control), resulting in facilitated processing at the position of the salient stimulus (Jonides, 1981). Singletons are stimuli with at least one feature that separates them from their feature-homogenous surrounding stimuli, for example, a green apple among red apples.

While the bottom-up influences on attentional guidance originate from the stimuli themselves (exogenous influences), top-down processes refer to influences on attentional guidance from within the organism. A prime example of a top-down process is the ability to voluntarily shift what is subjectively perceived as spatial attention towards a specific location without moving the eyes (Posner, 1980; von Helmholtz, 1894). The same top-down control of spatial attention, however, can also be based on top-down templates (i.e., attentional control settings or search criteria) for target-defining features by which the visual field is then searched in parallel (Andersen et al., 2008; Andersen et al., 2009; Bichot et al., 2005; Desimone & Duncan, 1995). All these top-down processes can trigger a shift of attention based on a participant’s prior voluntary decision to direct attention to a particular location or to look for a particular feature. Note that a voluntarily chosen search criterion (e.g., a decision to look for the target-defining color) also influences attentional guidance involuntarily, even if the criterion-matching stimulus is (partly) irrelevant. First, if participants search for a red target, all red stimuli involuntarily capture attention, regardless of whether they are targets or irrelevant target-preceding cues (Folk et al., 1992; Folk & Remington, 1998; Lien, Ruthruff, & Cornett, 2010). Second, this indirect top-down influence on attentional guidance interacts with bottom-up processes, sometimes preventing attentional capture by salient stimuli that do not match the search criterion altogether (Ansorge et al., 2011; Eimer & Kiss, 2008). The influences of bottom-up and top-down processes have sometimes been assumed to converge on a single priority map, in which attentional priority is projected to different degrees on different areas of the visual field. Based on this priority map, attention would then be oriented towards locations with high priority (cf. Wolfe, 1994, 2007, 2021).

Importantly, there is an assumed third influence on attentional guidance: Selection history can alter visual search performance, and its influence can neither be attributed to that of salience nor that of top-down search criteria alone (cf. Awh et al., 2012). Selection history refers to the influence of previous experiences and encompasses a broad range of phenomena like priming (e.g., Kristjánsson & Ásgeirsson, 2019; Maljkovic & Nakayama, 1994, 1996), statistical learning (e.g., Gao & Theeuwes, 2020; Geng & Behrmann, 2005; Wang & Theeuwes, 2018a, 2018b), contextual cueing (e.g., Chun & Jiang, 1998, 2003), and (reward-based) learning (e.g., Anderson, 2013; Anderson et al., 2011; Feldmann-Wüstefeld et al., 2015; Kim & Anderson, 2019). Despite this heterogeneity, selection history boils down to the influences of implicit or explicit memory on visual search performance and attentional guidance (cf. Awh et al., 2012; Wolfe, 2021; Wolfe & Horowitz, 2017).

To note, however, the influence of selection history on visual search performance does not necessarily mean it influences attentional guidance as well (e.g., Becker & Ansorge, 2013; Johnson et al., 2020; Kinchla et al., 1995; Shiu & Pashler, 1994). For example, improved search performance could also result from response-related or perceptual facilitation after attention is already oriented towards a location (Ramgir & Lamy, 2021). Indeed, recent studies found ambiguous results regarding the influence of selection history on attentional guidance. For example, Ramgir and Lamy (2021) comprehensively reviewed intertrial priming results and found only inconclusive evidence for an influence on attentional guidance. Luque et al. (2021) investigated contextual cueing and concluded that contextual cueing produces perceptual learning processes instead of influencing attentional guidance (but see, e.g., Harris & Remington, 2017). Jiang et al. (2015) found only a negligible influence of reward learning on attentional guidance.

Therefore, the conditions under which different instances of selection history influence attentional guidance warrant further investigation. Persistent attentional guidance based on selection history would limit how flexibly humans can ignore hitherto relevant but now irrelevant information and focus on currently relevant information instead (cf. Wenke et al., 2009). Here, we address these questions by investigating whether a feature previously learned as target-defining influences attentional guidance and may even persist in doing so during a later task where that feature is no longer target defining.

To measure attentional guidance, we used the contingent-capture design, a modified spatial cueing protocol introduced by Folk et al. (1992), and combined it with a trial-and-error learning task. Participants responded to one of four different stimuli (possible targets) in the target display and received positive or negative feedback after each response for correct or wrong target selections, respectively. Before each target display, a salient singleton cue appeared randomly at one of the four possible stimulus positions. This cue was nonpredictive and, hence, appeared in 25% of the trials at the target position (valid trials) and 75% at a nontarget position (invalid trials). Attentional capture of the cue is measured by subtracting the mean reaction times in valid trials from those in invalid trials. If a cue captures attention, visual processing is facilitated at the cue position, and, thus, responses to a target appearing at the cued position (valid trial) are faster than in trials where the cue appeared at a nontarget position (invalid trials). This difference is called the validity effect, which is positive if a cue captures attention.

Conversely, if a cue does not capture attention, visual processing is not facilitated at the cued position, and there is no difference in reaction times between valid and invalid trials (resulting in a validity effect of zero). Finally, a negative validity effect would indicate that the cue delayed visual processing at its position (cf. Forstinger et al., 2022; Lamy et al., 2004).

The contingent-capture protocol has a few advantages over visual search tasks without a cueing display. First, the cue is presented among homogenous nonsingleton stimuli and temporally separated from the target display. Thus, its influence on attentional capture is far less susceptible to target–distractor similarities and possible distractor suppression processes, which would confound the measure of attentional guidance by the cue. Second—related to the temporal distance between cueing and target display—it is easy to use different cues to probe the attentional control settings without interfering with the target–distractor similarities (the relationship between target and distractors can influence the task, and, thus, the attentional control settings). Finally, the validity effect directly measures spatial guidance of attention (since it is calculated as the reaction time difference between valid and invalid trials). Nonspatial influences like filter costs or response-related processes might influence reaction times but would do so in valid and invalid trials and, thus do not influence the validity effect. Therefore, the validity effect reflects a spatially selective process of attention guidance or capture.

## Experiment 1

In Experiment 1, participants had to learn the target color in the first block (color block). Then, in the second block, we instructed them to search for a specific orientation as the target-defining feature. This procedure allows us to investigate whether a possible attentional bias towards the learned target color persists after a different target feature is instructed. If the selection history of the previously learned and used target color biases attentional guidance independent of currently used search criteria, we expected that the learned target color would capture attention at least for some time, even after the participants search for a new target feature and the previous target color has become task-irrelevant. Otherwise, if the currently used search criterion dominates attentional guidance, the previously target-defining color should stop biasing attention once participants start using a new search criterion.

Independent of selection history, we expect that—after participants know the target feature—cues matching that target feature capture attention more than nonmatching cues do. Throughout the experiment, we used four different cues. One cue matched the target color in the color block and the target orientation in the orientation block. Two other cues matched only the target color (in the color block) or only the target orientation (in the orientation block). The fourth cue matched neither the target color nor the target orientation.

### Method

#### Participants

In all experiments, we decided in advance on a sample size of between 20 and 25 participants. We tested at least 20 participants but did not send away already registered participants if the sample size was still smaller than 25. According to classical power analysis (e.g., G*Power; Faul et al., 2007), a sample of 20 participants results in a power of .97 to find an effect of Cohen’s *d* = 0.9 (for a two-sided one-sample *t* test with a significance level of 𝛼 = .05). The effect size of 0.9 is about half of the mean effect size of contingent-capture effects reported in a recent meta-analysis (Büsel et al., 2020).

Twenty-one participants (15 women, six men) aged between 18 and 30 years (*M* = 20.90, *SD* = 2.61, *Mdn* = 20) took part in this experiment. Here and in the following experiments, all participants had self-reported normal or corrected-to-normal visual acuity, no red–green deficiency (examined with Ishihara color plates), and gave written informed consent before the experiment. In all experiments, participants received course credits for their participation and were treated in compliance with the ethical standards of the Declaration of Helsinki as well as the national and institutional ethical standards.

#### Apparatus

The participants sat in a dimly lit room in front of an LCD monitor. The monitor (AOC Gaming Monitor G2590PX, 24.5 in) had a resolution was 1,920 × 1,080 pixels (54.4 × 30.3 cm) and a refresh rate 100 Hz. A chin rest assured a viewing distance of 57 cm to the center of the monitor. We used MATLAB (The MathWorks, Inc., Natick, Massachusetts, USA) version 9.6 (R2019a) and the Psychtoolbox 3.0.15 (Brainard, 1997) to program and control the experiment.

#### Design and procedure

The design consisted of three independent within-subject variables (cue condition [color and orientation matching vs. color matching and orientation nonmatching vs. color nonmatching and orientation matching vs. color and orientation nonmatching], validity [valid vs. invalid], target feature [color vs. orientation]). Participants watched 15 illustration trials before data collection and had to learn the target feature. To this end, each stimulus in the target display was centered on a white circle with a gap at the top, bottom, left, or right. Participants had to report the gap position by pressing the corresponding arrow key of a standard computer keyboard. After each response, they received feedback (“Richtig! [Correct!]” or “Falsch! [Wrong!]”) to indicate whether they correctly chose the target or not. Through this feedback, the participants learned the target via trial and error. After they reached an accuracy of at least 75% during the last 20 trials,^1^ a dialog box popped up where they typed in the discovered rule to find the target and proceeded with the experiment. After the first block, in which the color red defined the target (color block), the second block followed, in which the horizontal orientation defined the target (orientation block). Participants were instructed to search for the horizontal line at the beginning of the second block. Each block consisted of 768 trials (48 valid and 144 invalid trials per cue condition). The trials were presented in bins of 64 trials with 16 valid and 48 invalid trials (four valid trials per cue condition). The order of trials in each 64-trial bin and the order of bins was pseudorandomized. This procedure ensured that valid trials of each cue condition occurred fairly uniformly across the experiment. There were five breaks (two within each block and one between the blocks) which the participants could terminate by pressing the space bar. Including the illustration trials and breaks, the experiment lasted ca. 75 min.

**Fig. 1:**
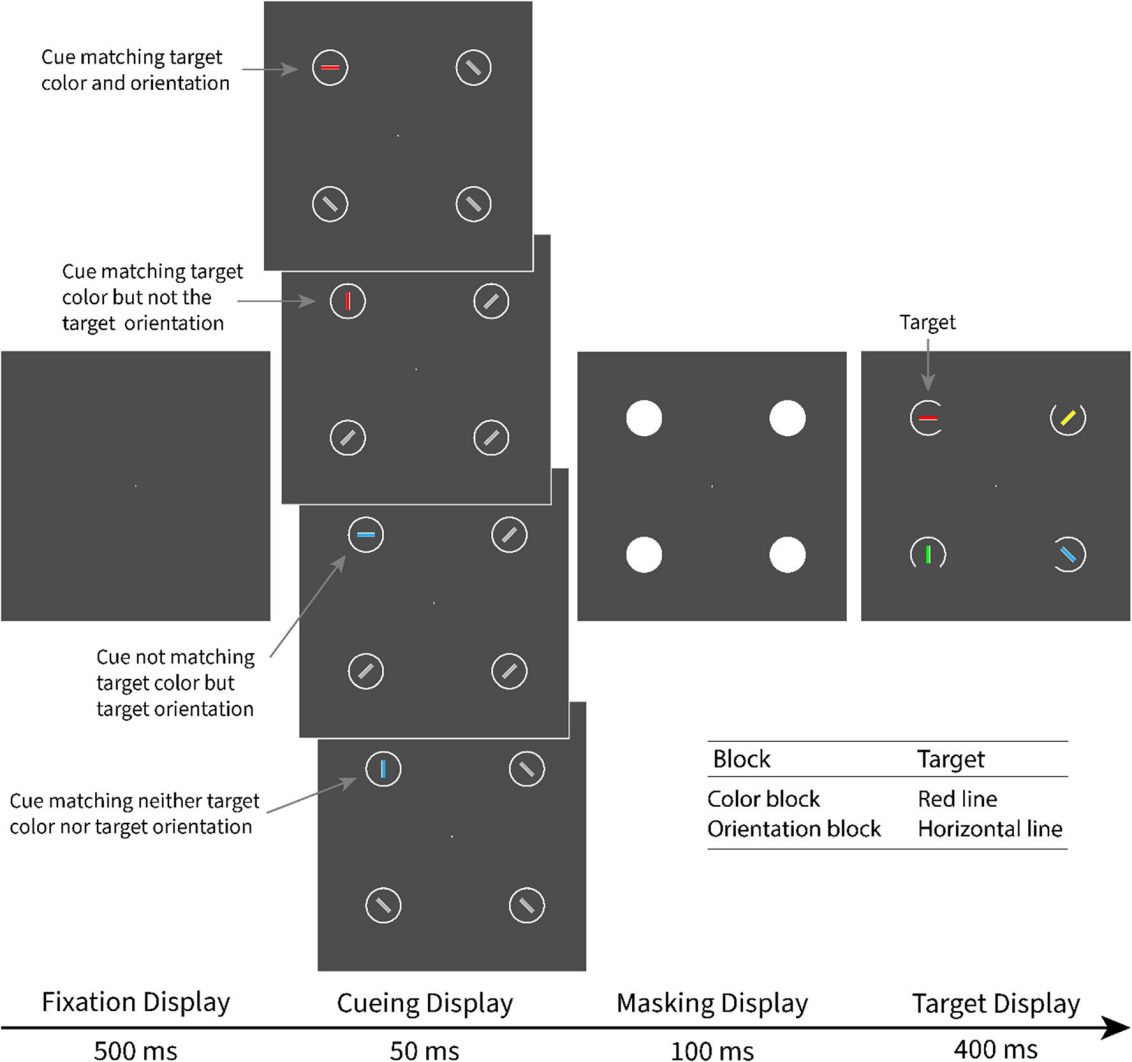
Procedure of Experiments 1 and 2. This figure depicts a valid trial with all cue conditions for the red target in the color block and the horizontal target in the orientation block. Not depicted is the response display (shown for 1 s or until response) and the feedback display (shown for 1 s). Both followed the target display. The stimuli are drawn to scale, but the gray background is cropped and does not represent the screen size. (Color figure online)

Each trial started with a fixation display, consisting of a white point on a dark-gray background at the center of the screen. After 500 ms, the cueing display was presented for 50 ms. The cueing display consisted of four lines. One line was the singleton cue, which was either a red horizontal line (color and orientation matching), a red vertical line (color matching and orientation nonmatching), a blue horizontal line (color nonmatching and orientation matching), or a blue vertical line (color and orientation nonmatching). Since the cues were kept the same in both blocks, we consistently use these condition labels even though the matching of a feature refers only to the block in which that feature was target defining. Thus, a red cue is denoted as color matching, regardless of whether it is presented in the color block or the orientation block. For example, the red cue did not match a search criterion for the target-defining feature in the orientation block anymore. This labeling helps distinguish cues matching a top-down search criterion in one of the blocks from the nonmatching cues that never matched the top-down search criterion. The other lines in the cueing display were nonsingletons and always all gray and either all 45° tilted to the left or all 45° tilted to the right. The target display consisted of four lines in four colors (red, green, blue, and yellow) and four orientations (horizontal, vertical, 45° tilted to the left, and 45° tilted to the right). See Fig. 1 for examples of all types of displays and conditions.

In a variant of the experiment, the target was defined as the green line in the color block and the vertical line in the orientation block. Accordingly, the red cue color was changed to green, and the roles of horizontal lines versus vertical lines as matching versus nonmatching cues were reversed. Otherwise, all stimuli and the procedure were the same. Participants were randomly assigned to a variant of the experiment.

#### Stimuli

The stimuli were presented at the corners of an imaginary square at the center of the screen. The horizontal and vertical offset from the center was 6.42° of visual angle. The background of the display was dark gray (CIELAB color space: L* = 35, a* = 0, b* = 0), the noncolored rings were white (L* = 140, a* = 0, b* = 0), and the disks inside the rings were medium gray (L* = 70, a* = 0, b* = 0). The colors of the target-display disks were yellow (L* = 70, a* = 0, b* = 73), red (L* = 70, a* = 99, b* = 90), green (L* = 70, a* = −70, b* = 67), and blue L* = 70, a* = 25, b* = −110). The color of the nonmatching cue was blue. The white fixation point had a diameter of 0.2° visual angle. The placeholders were white circles with a diameter of 3.38° and a line width of 0.14°. The masking disk was white and covered the placeholder. The lines were 1.69° long and 0.28° thick. On the long side of the lines, a thin (0.04°) white outline accentuated the line. We chose to add this outline to provide the lines with a color-independent feature that indicates their orientation. The gap size in the target placeholders was 2.25°.

#### Data analysis

We used R (Version 4.1.3; R Core Team, 2021) and the R-packages *data.table* (Version 1.14.2; Dowle & Srinivasan, 2020), *emmeans* (Version 1.7.2; Lenth, 2019), *ggplot2* (Version 3.3.5; Wickham, 2016), *lme4* (Version 1.1.28; Bates et al., 2015), *outliers* (Version 0.14; Komsta, 2011), and *psychometric* (Version 2.3; Fletcher, 2010) for our analyses. We analyzed only reaction times between 150 ms and 1 s of correctly answered trials for the validity effect analysis. The validity effect was our primary indicator of attentional guidance. We calculated a validity effect per participant for all cue conditions and tested whether validity effects or validity effect differences differed from zero or from each other using two-sided, one-sample *t* tests. For all analyses, we used a significance level of 𝛼 = .05.

Furthermore, we analyzed the validity effect over time to see how attentional guidance develops after participants learned the target-defining feature and after the learned target-defining feature changes. To this end, we calculated the validity effect for each trial sequence of 64 trials (i.e., per bin) per cue condition and participant. Each bin consisted of four valid trials and 12 invalid trials per cue condition. Since the validity effect is the difference between mean reaction times in valid compared with invalid trials, the number of reaction times used to calculate the means is crucial for the reliability of the validity effect. The four valid trials per cue condition and bin are already very low. Thus, using a smaller bin size—which would further decrease the number of valid trials—is not reasonable. Therefore, validity effects over time cannot be analyzed with an arbitrary temporal resolution, and the bin size we used is near the highest temporal resolution that is reasonable.

Finally, we analyzed the accuracy rate (proportion of correct responses during the last 20 trials) over time to relate the validity effects to the performance in the search task. First, we derived the trial in which a participant learned the target-defining feature based on the number of correct answers during the previous 20 trials. If this number reached 16 (80%), we inferred that the participant had learned the target-defining feature 19 trials prior since one would need about 19 trials to reach that criterion starting from chance level accuracy (25%)—assuming a general error rate of 10% after learning the target-defining feature.

Then, we defined the trial in which the target-defining feature was learned as trial zero, with previous trials having negative numbers and following trials having positive numbers, and averaged the accuracy rate across participants for each trial number. This procedure to analyze the learning process is necessary since averaging the individual learning curves might result in a learning curve (vastly) different from any actual learning curve (cf. Estes, 1956; Hayes, 1953). Note that the amount of negative and positive trial numbers (trials prior to and after learning) varies among participants depending on the point in time when they learned the target feature. Therefore, the average accuracy rate in the most negative trials is based on only a few participants, and we did not plot values in trials with less than four data points.

The validity effects were averaged across participants for each bin centered around the trial where the target-defining feature was learned. If that trial (trial zero) occurred within a bin, the validity effect within this bin was calculated separately for trials before and after learning. Therefore, we avoided artificial carryover of validity effects before and after learning the target-defining feature. If a participant had two or fewer correct reaction times in a bin, we did not calculate a validity effect for this bin. Additionally, if we had two or fewer validity effects per trial across participants, we removed the validity effect for these trials from our analysis. Thus, the validity effects of some trials are not depicted in Fig. 3.

### Results

For the validity effect analysis, we excluded all incorrectly answered trials and trials before participants reached the learning criterion of 80% accuracy (11.04% for the color block and 29.62% for the orientation block). From the remaining trials, we excluded trials with reaction times below 150 ms and above 1 s (0.03% for the color block and 0.19% for the orientation block). After the exclusions, the average number of valid trials per experimental condition ranged from 34.81 to 43.24. The average Intraclass Correlation 1 (ICC1) within experimental conditions was 0.19, and the Intraclass Correlation 2 (ICC2) was .94 in the color block. In the orientation block, the average ICC1 was .09, and the ICC2 was .84. Across all conditions, the ICC1 was .17, and the ICC2 was .99 in the color block (.07 and .98 in the orientation block, respectively), indicating sufficient measurement reliability in all conditions.

#### Validity effects

**Fig. 2:**
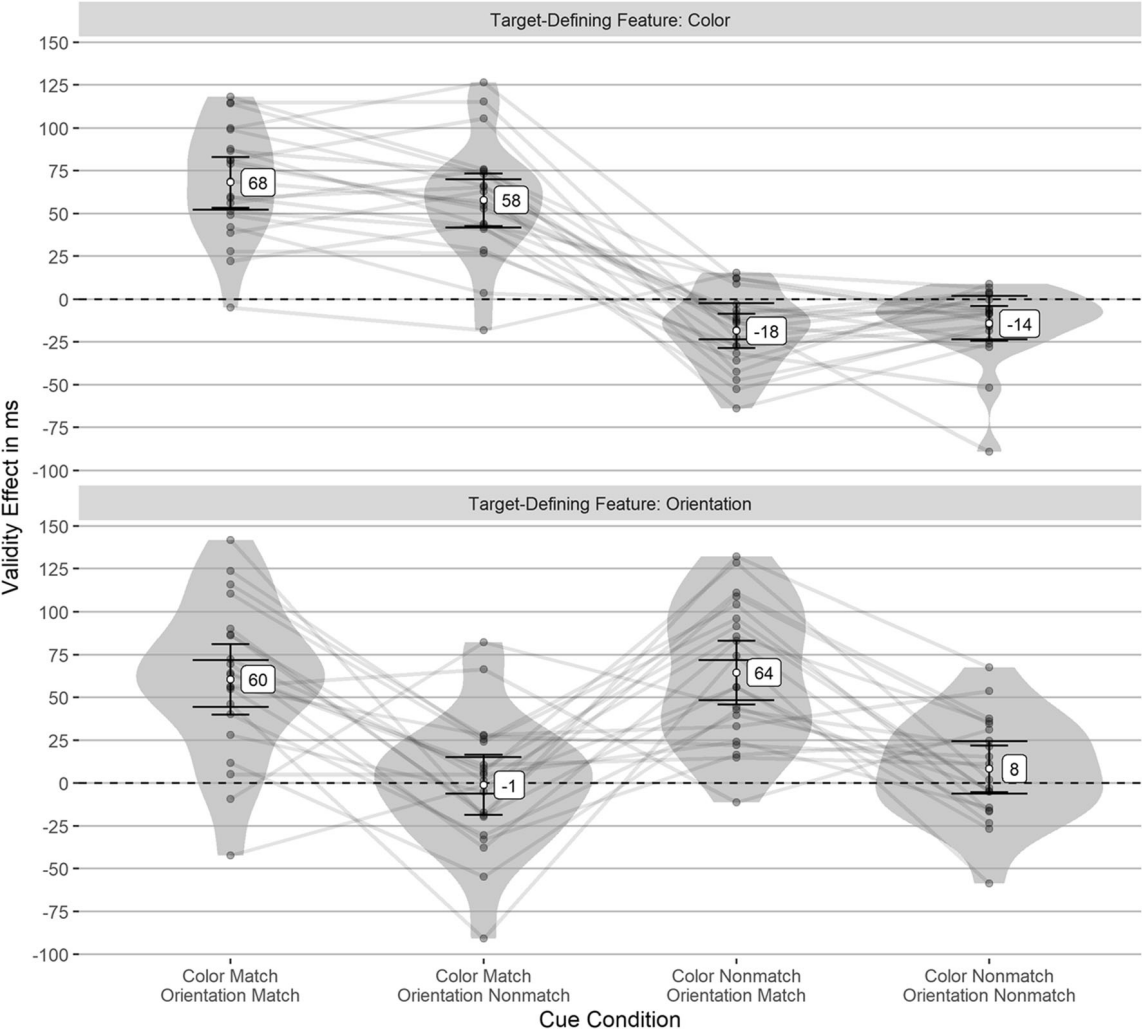
Validity effects (following learning in color block) in Experiment 1. This figure depicts the validity effects for all cue conditions in each block. The individual validity effects (gray points) are plotted to show their distribution. The gray lines connect values of the same participant, and the narrow black error bars represent the 95% CI for the one-sample *t* test against zero. The wide error bars represent the 95% CI for the difference between the conditions in each block. No overlapping indicates a significant difference

According to Shapiro–Wilk tests, the validity effects were normally distributed in all but one experimental condition. However, in this condition, a Wilcoxon signed-rank test yielded the same results as the *t* tests. Therefore, we used *t* tests for consistency. The results showed strong significant validity effects only for cues matching the current target-defining feature. Cues without such a feature showed significant but small negative validity effects in the color block and a nonsignificant validity effect in the orientation block (see Table 1). Figure 2 shows the validity effects. The wide error bars indicate whether there is a significant difference between cue conditions.^2^ The reaction times are shown in Fig. 11 in the Appendix. Simulations to estimate the achieved power (cf. Arnold et al., 2011) indicated a power of 88% in the color block and 82.1% in the orientation block to find a validity effect of 25 ms as significant above zero (21 participants, *α* = .05). We chose these effect sizes independently and not based the actual effect sizes in Experiment 1 to avoid the misleading practice of reporting post-hoc power for the effect size found in the experiment (cf. O’Keefe, 2007). The rational for using simulation to estimate achieved power is described in more detail in the appendix of Grüner et al. (2021). In all experiments, we simulated reaction times from the actual reaction time distribution in each experiment. The sample size and number of trials per participant were the same as in the real experiment (to account for the excluded trials and the unbalanced design).

**Table 1:** Mean validity effects (following learning in the first block) in Experiment 1

Cue Condition	*M* (*SD*)	95% CI	*t*(*df*)	*p* ^**a**^	*d*_*unb*_ ^**b**^	95% CI
Target feature: Color						
Color match, Orientation match	68 (33)	[53, 83]	9.53(20)	<.001	2	[1.3, 2.84]
Color match, Orientation nonmatch	58 (34)	[42, 73]	7.81(20)	<.001	1.64	[1.02, 2.37]
Color nonmatch, Orientation match	−18 (22)	[−29, −8]	−3.84(20)	.004	−0.81	[−1.33, −0.33]
Color nonmatch, Orientation nonmatch	−14 (22)	[−24, −4]	−2.94(20)	.03	−0.62	[−1.11, −0.16]
Target feature: Orientation						
Color match, Orientation match	60 (45)	[40, 81]	6.1(20)	<.001	1.28	[0.73, 1.91]
Color match, Orientation nonmatch	−1 (39)	[−19, 16]	−0.14(20)	1.00	−0.03	[−0.46, 0.4]
Color nonmatch, Orientation match	64 (41)	[46, 83]	7.2(20)	<.001	1.51	[0.92, 2.21]
Color nonmatch, Orientation nonmatch	8 (30)	[−5, 22]	1.27(20)	.68	0.27	[−0.16, 0.71]

Mean and *SD* in ms. The feature match is only applicable in the corresponding target-feature block. That is, there is no matching color when orientation defines the target. In this case, matching refers to the previous block, where the color was target defining

^a^Corrected for multiple comparisons using the method of Benjamini and Yekutieli (2001)

^b^Effect size standardized with the group *SD* and corrected using Hedges’s correction factor (Hedges, 1981)

#### Learning curve and validity effects over time

**Fig. 3:**
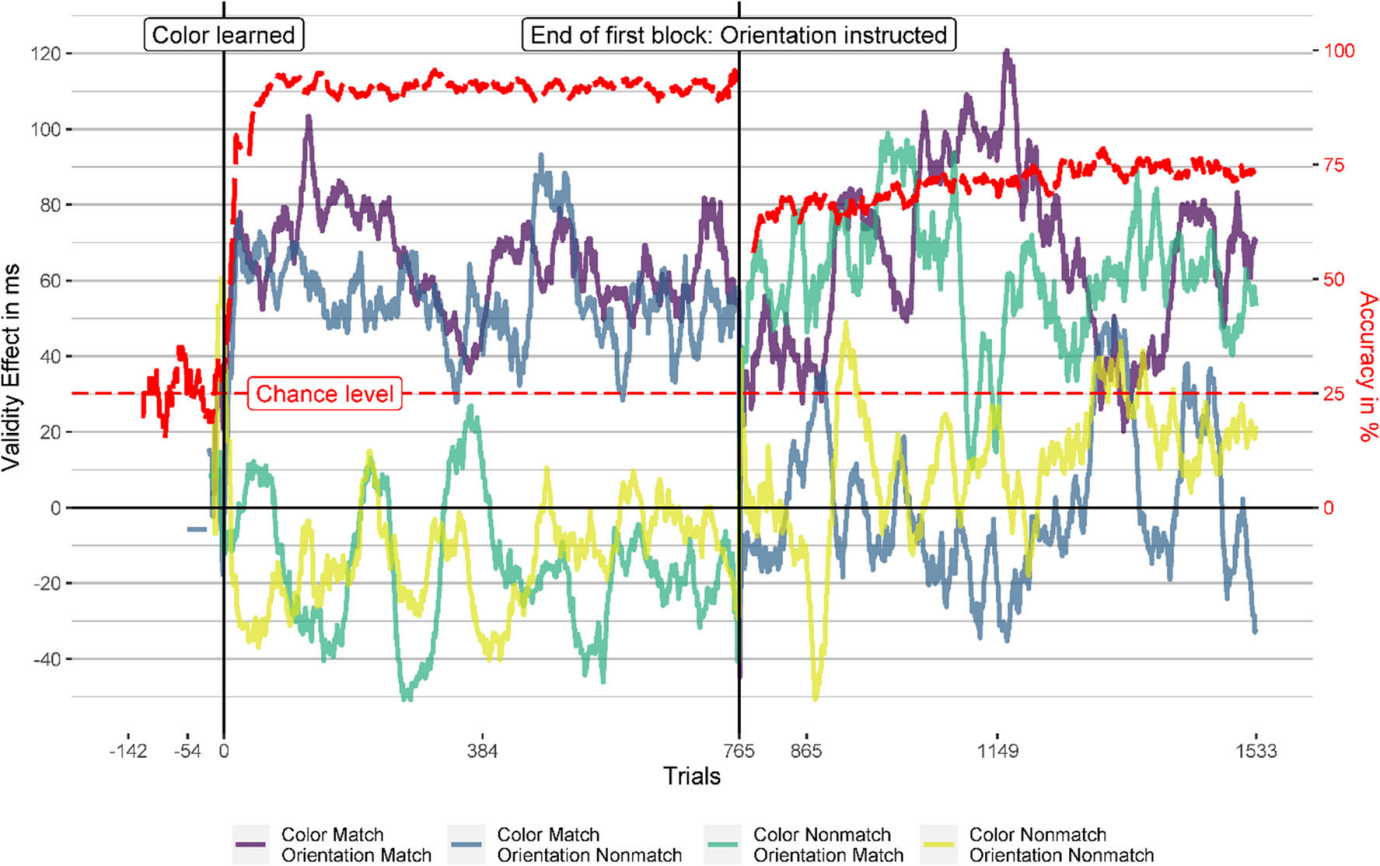
Learning curve and validity effects in Experiment 1. This figure depicts the accuracy (red, dashed line) and validity effects over time for all cue conditions. The vertical lines mark the trial where the participant learned the target feature (left vertical line) or where they were instructed to search for orientation (right vertical line). The validity-effect and accuracy lines are smoothed using a moving average of 20 trials and five trials, respectively. See the Results section of Experiment 1 for further explanation. (Color figure online)

All participants learned the target color (i.e., reached 80% accuracy or more in the last 20 trials) after a maximum of 142 trials (*Mdn* = 15). Figure 3 shows the accuracy and response validity effects over time for the learning/color (first block) and instruction/orientation (second block) blocks. The vertical lines mark the time point of learning the target feature (left vertical line) and the end of Block 1 (right vertical line), where we instructed the participants to search for the target orientation.

In Block 1, the results indicate that the participants learned the target features almost at once, in a relatively small number of successive trials, probably through insight, and that the validity effects of cues similar to the target-defining feature also developed fast after learning the target feature. After instruction to search for the new target-defining feature (orientation), the now matching orientation cues quickly elicited validity effects. Equally fast, the validity effect in the matching color cue conditions vanished after participants were told that the target-defining feature is the orientation and not the color anymore (at the end of the first block). The cue with the new target-defining feature in the orientation block elicited a validity effect similar to that in the color block, despite a drop in accuracy during the orientation search.

**Fig. 4:**
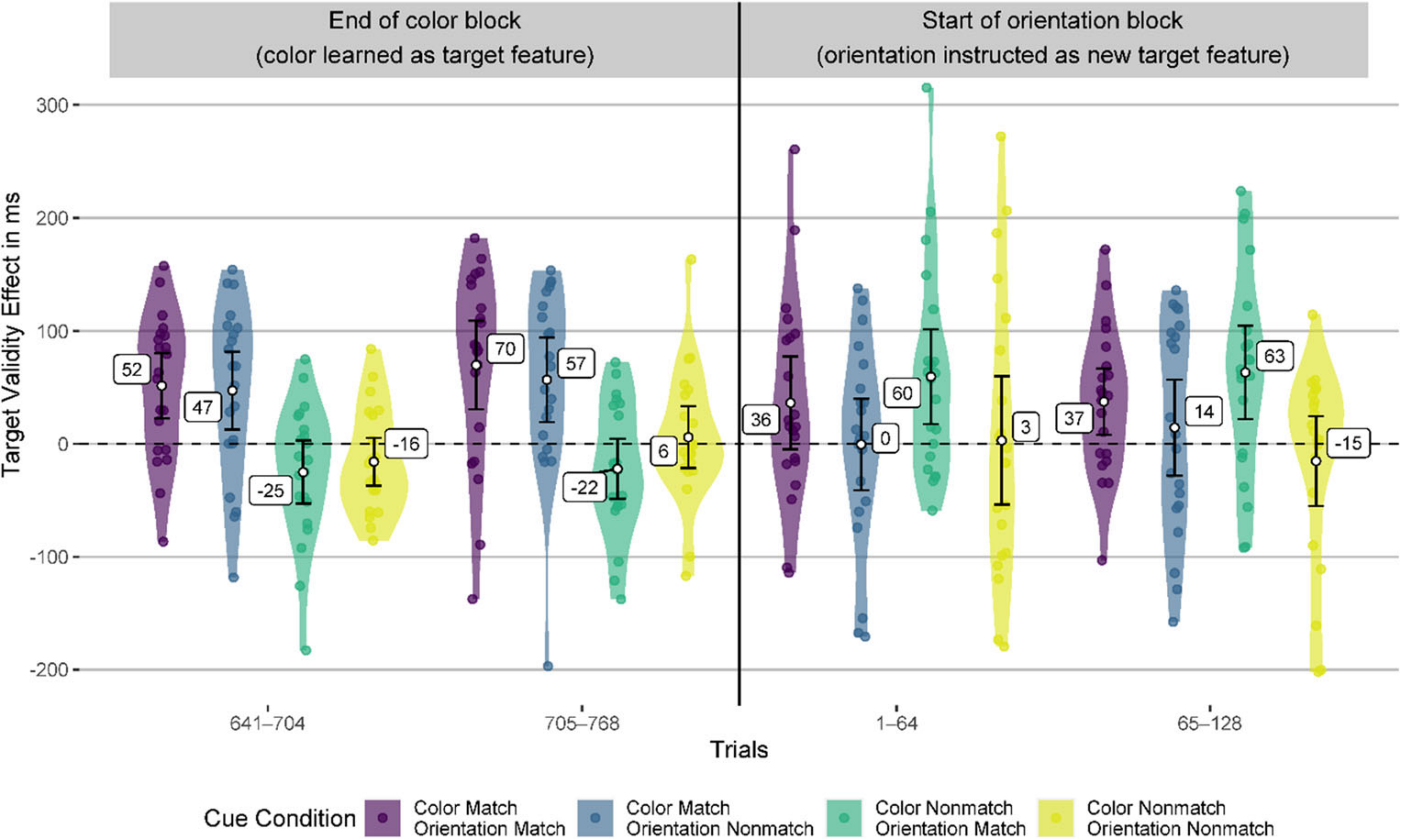
Validity effects in Experiment 1, before and after the target feature changed. This figure depicts the validity effects in the two bins (64-trial sequences) before and after the target feature changed. The error bars represent the 95% CI from the one-sample *t* test against zero. (Color figure online)

In Fig. 3, the smoothing might disguise a potential attentional capture of cues matching the previous target color in the first trials of the orientation block. Therefore, we analyzed the last two bins of the color block and the first two bins of the orientation block separately. Each bin consisted of 64 trials, which is the smallest bin size that can yield at least somewhat reliable validity effects (see section Data Analysis). We found that in the two last bins of the color block, cues with the target-defining feature (color) elicited significant validity effects (47 to 70 ms, all *p*s < .009), while the cues with a different color did not elicit significant validity effect (−25 to 6 ms, all *p*s > .076). After the target-defining feature changed to orientation in the second block, cues with the target orientation elicited validity effects of a similar magnitude as the cues with the target color in the previous block already in the first bin of the second block (36 to 63 ms). These validity effects were significantly above zero (*p*s < .015) except for the cue with matching color and orientation in the first bin (*p* = .081). Importantly, the cue with the previous target color and a nonmatching orientation elicited a validity effect of zero (*p* = .98) in the first bin of the orientation block and 14 ms (*p* = .49) in the second bin, indicating no attentional guidance by a previous but now irrelevant target feature. Figure 4 shows the validity effects in the two bins before and after the target-defining feature changed and Table 5 in the Appendix shows the corresponding statistical information, CIs, and effect sizes.

### Discussion

After the participants learned the target-defining feature, their accuracy increased quickly to the maximum. The validity effects occurred immediately after the target feature was learned and were contingent on the cue’s resemblance to the target-defining feature. After getting instructed to search for the orientation, the validity effect of the cue matching only the previously target-defining feature dropped to zero within the first 64 trials. At the same time, cues matching the new target feature (orientation) started capturing attention.

These results indicate that a potential influence of a learned and selected target-defining feature on attentional guidance vanished within 64 trials once participants were instructed that this learned feature was no longer task relevant. Participants flexibly and swiftly adjusted their attentional control setting to the instructed new search criterion (cf. Lien, Ruthruff, & Johnston, 2010) without evidence for a lingering attentional bias toward a previously selected feature. Since the temporal resolution of the validity effect measurements is unavoidably limited, we cannot rule out such a bias within the first 64 trials. However, capture by the previously matching cue vanished at the same time course as the currently matching cue started capturing attention, indicating that possible influences of selection history do not last longer than the time needed for new attentional control settings to emerge.

However, the instruction to search for the new target-defining feature might have concealed a possible influence of selection history on attentional guidance since the previous target feature was incompatible with the new target feature. That is, each cue simultaneously carried a relevant and an irrelevant feature (a specific orientation and a particular color, respectively), and using orientation might overwrite any selection history effect of color. Additionally, the previous target color appeared more often on a distractor than on the new target, which might encourage the suppression of the distractor-associated feature despite a potential bias due to selection history.

To account for this possibility, in Experiment 2, we repeated Experiment 1 but instructed participants to look for a novel target-defining feature and relearn the search criterion in the second block instead of instructing them to search for orientation. Hence, there will be a period after the first block where participants are only instructed that the previous target-defining feature is no longer target-defining, but they do not yet know the new target-defining feature. During this period of uncertainty, possible influences of selection history are not influenced by attentional control settings for the new target-defining feature (since it is still unknown).

## Experiment 2

In Experiment 2, we repeated Experiment 1 with one change: Instead of instructing the participants to search for the new target-defining feature (orientation), we instructed them that the target is no longer defined by its color (as in the first block) and that they have to learn the new target-defining feature (which was the same orientation as in Experiment 1). If a potential influence of selection history were disguised due to the attentional control setting established following the instruction to search for a particular alternative feature in Experiment 1, we would expect to find an influence of selection history on attentional guidance during the time when participants have not yet learned the new target-defining feature. Furthermore, we expected to replicate the findings of Bock 2 in Experiment 1 after participants learned the new target-defining feature.

### Method

Twenty-two participants (18 women, four men) aged between 18 and 26 years (*M* = 20.73, *SD* = 1.93, *Mdn* = 20.50) took part in Experiment 2. All participants were tested under the same general conditions as in Experiment 1. Furthermore, except for the different instructions at the end of Block 1, the design, apparatus, and stimuli were the same as in Experiment 1 (see Fig. 1).

#### Data analysis

As in Experiment 1, we calculated the validity effect for bins with 64 trials each. However, during the time when participants have not yet learned the new target-defining feature in the second block, we would have to exclude most of the trials as incorrect trials (performance prior to learning is at chance level [25% accuracy]). Thus, on average, we would end up with only one correctly answered valid trial per cue condition from four such trials occurring per bin—which does not allow calculating reliable validity effects. Therefore, relying on correct answers is very problematic during the time when we expect possible effects of selection history: when participants have not yet set up an attentional control setting for the new target, which could potentially replace the one for the previous target features. The measurement reliability would be too low to find an effect reliably.

However, since each stimulus in the target display was associated with a unique response, we could determine to which stimulus participants responded. Therefore, we kept incorrectly answered trials and calculated the mean reaction time differences between responses to validly cued stimuli (appearing at the same position as the cue) and invalidly cued stimuli (appearing at a different position than the cue). We refer to this measure as the response validity effect since it is similar to the usual validity effect but is not restricted to correct responses and refers to cueing of just any stimulus to which a participant responded, not to the targets only. If a cue captures attention based on a previously relevant feature, reaction times to stimuli at the cued position (valid trials) should still be faster than to stimuli at uncued positions (invalid trials). The advantage of this procedure is that we can calculate a reliable response validity effect even when participants performed at a chance level (e.g., when they do not know the target-defining feature yet). Additionally, if participants responded correctly, the response validity effect is the same as the usual validity effect.

### Results

We excluded 7.06% of trials with reaction times below 150 ms and above 1 s (including trials where no response was given) from all analyses. For the validity effect analysis, we excluded data from one participant who did not learn the target feature in both blocks and three participants who did not learn the target feature in the second block. From the remaining data, we excluded all incorrectly answered trials and trials before participants reached the learning criterion of 80% accuracy (13.47% for the color block and 40.89% for the orientation block) and then removed trials with reaction times below 150 ms and above 1 s (no trials in the color block and 0.07% in the orientation block). Due to the relatively high number of excluded trials in the orientation block, 30 (*SD* = 11) valid trials remained on average. Consequently, measurement reliability suffered in the orientation block but was still acceptable (ICC1 = .04 and ICC2 = .94 for the entire data set, and an average of ICC1 = .06 and ICC2 = .71 across conditions). All other ICC1s and ICC2s were a least .13 and .92, respectively.

#### Validity effects after learning the target-defining feature

**Fig. 5:**
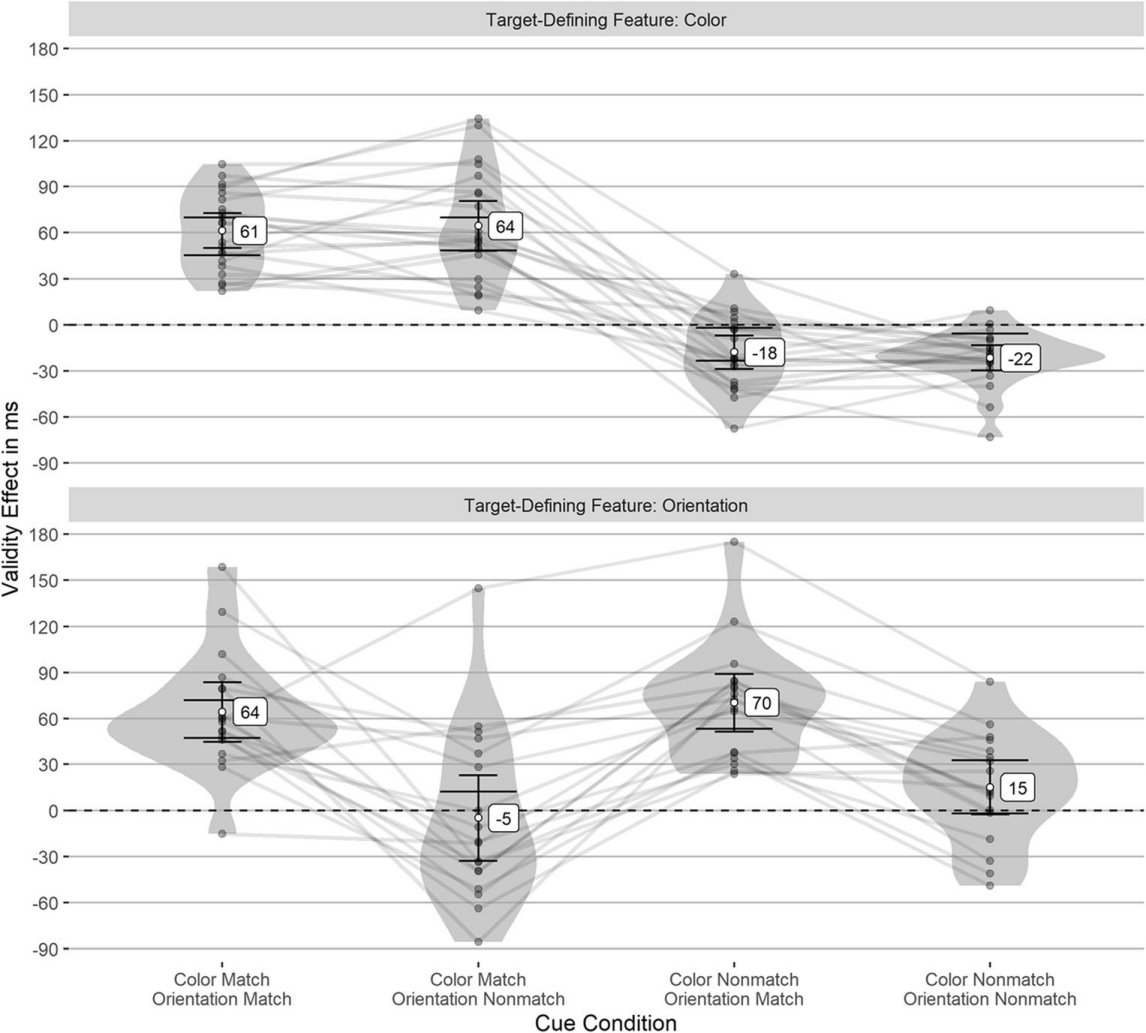
Validity effects (following learning in both blocks) in Experiment 2. This figure depicts the validity effects for all cue conditions in each block. The individual validity effects (gray points) are plotted to show their distribution. The gray lines connect values of the same participant, and the narrow black error bars represent the 95% CI for the one-sample *t* test against zero. The wide error bars represent the 95% CI for the difference between the conditions in each block. No overlapping indicates a significant difference

Validity effects in all but two conditions were normally distributed, but we used only *t* tests since the results would be the same as with nonparametric tests. The results showed strong significant validity effects only for cues matching the current target-defining feature. Cues without such a feature showed significant but small negative validity effects in the color block and a nonsignificant validity effect in the orientation block (see Table 2 and Fig. 5). The reaction times are shown in Fig. 12 in the Appendix. According to simulations, the achieved power was 98.7% in the color block and 71.8% in the orientation block to find a validity effect of 25 ms as significantly above zero (21 participants in the color block, 18 participants in the orientation block, *α* = .05). However, a validity effect of 30 ms (which is also on the smaller end of usually reported validity effects) is found with a power of 84.2% in the orientation block.

**Table 2:** Mean validity effects (after the target feature was learned) in Experiment 2

Cue Condition	*M* (*SD*)	95% CI	*t*(*df*)	*p* ^**a**^	*d*_*unb*_ ^**b**^	95% CI
Target feature: Color						
Color match, Orientation match	61 (25)	[50, 73]	11.17(20)	<.001	2.34	[1.57, 3.29]
Color match, Orientation nonmatch	64 (36)	[48, 81]	8.2(20)	<.001	1.72	[1.08, 2.48]
Color nonmatch, Orientation match	−18 (24)	[−29, −7]	−3.42(20)	.010	−0.72	[−1.23, −0.25]
Color nonmatch, Orientation nonmatch	−22 (18)	[−30, −13]	−5.46(20)	<.001	−1.15	[−1.75, −0.62]
Target feature: Orientation						
Color match, Orientation match	64 (39)	[45, 84]	6.97(17)	<.001	1.57	[0.92, 2.35]
Color match, Orientation nonmatch	−5 (56)	[−33, 23]	−0.37(17)	1.00	−0.08	[−0.55, 0.38]
Color nonmatch, Orientation match	70 (38)	[51, 89]	7.87(17)	<.001	1.77	[1.07, 2.62]
Color nonmatch, Orientation nonmatch	15 (35)	[−2, 33]	1.82(17)	.27	0.41	[−0.06, 0.91]

Mean and *SD* in ms. The feature match is only applicable in the corresponding target-feature block. That is, there is no matching color when orientation defines the target. In this case, matching refers to the previous block, where the color was target defining

^a^Corrected for multiple comparisons using the method of Benjamini and Yekutieli (2001)

^b^Effect size standardized with the group *SD* and corrected using Hedges’s correction factor (Hedges, 1981)

#### Learning curve and response validity effects

**Fig. 6:**
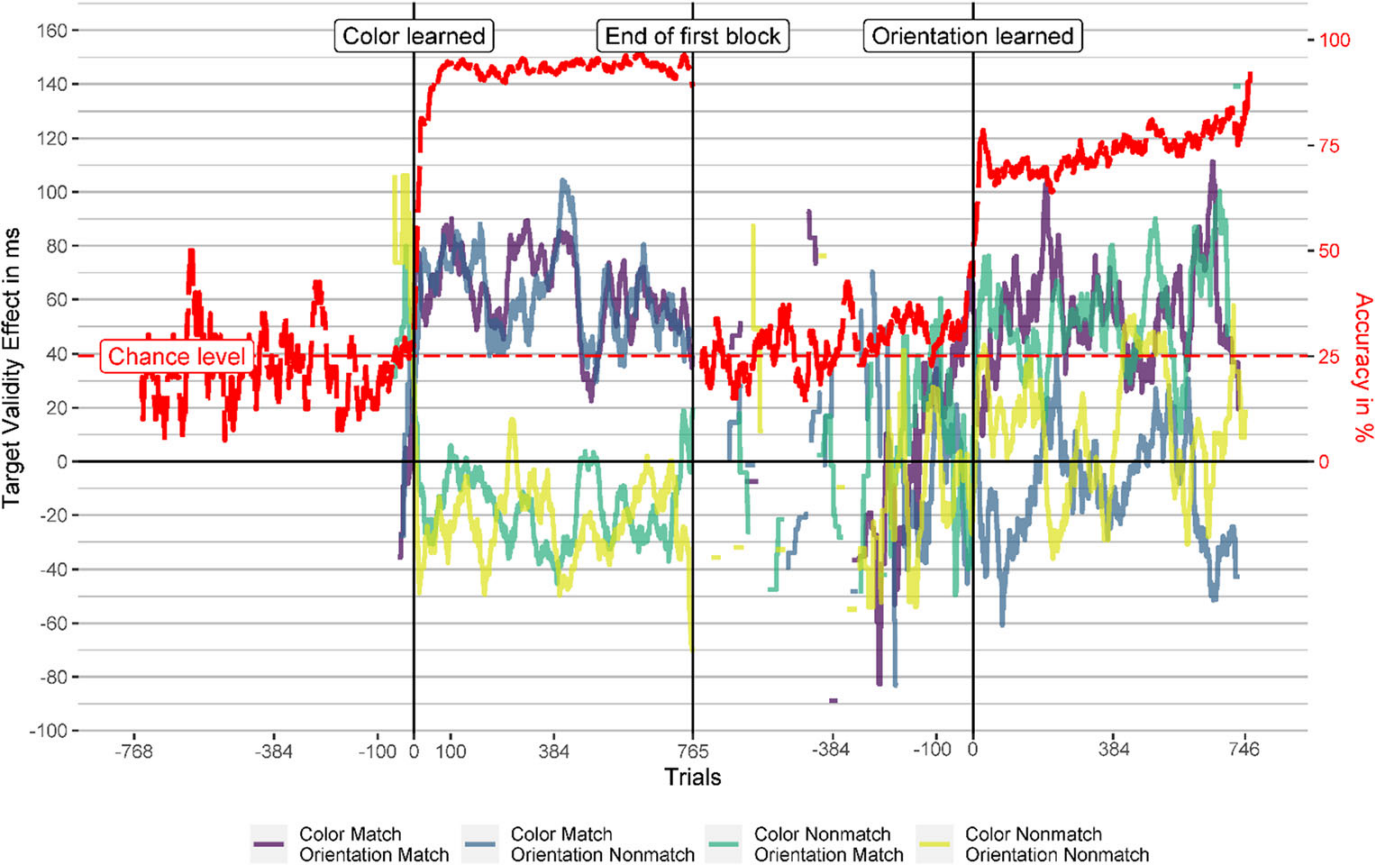
Learning curve and response validity effects in Experiment 2. This figure depicts the accuracy (red, dashed line) and response validity effects (reaction time to stimuli appearing at a different position than the cue minus reaction time to stimuli at the same position as the cue) over time for all cue conditions. The vertical lines mark the trial where the participant learned the target feature (left and right vertical lines) or where they were instructed to search for a novel target-defining feature (central vertical line). The validity effect and accuracy lines are smoothed using a moving average of 20 trials and five trials, respectively. See the Results section of Experiment 2 for further explanation. (Color figure online)

One participant did not learn the target feature in both blocks, and three participants did not learn the target orientation. The other participants learned the target color after a maximum of 516 trials (*Mdn* = 15) and the target orientation after a maximum of 668 trials (*Mdn* = 77.50). The learning criterion (80% accuracy or more in the last 20 trials) was the same as in Experiment 1. The accuracy and response validity effect over time was analyzed as in Experiment 1, and the results are shown in Fig. 6. However, this time participants had to learn two target features, and thus, we calculated two learning curves which we depicted in sequence in Fig. 6. Response validity effects based on two or fewer values are not depicted there, leading to missing data in the most negative trials. Using the response validity effect did not always alleviate that problem since some participants did not respond at all during many trials where they did not know the new target feature yet (perhaps in an attempt to look for possible noncolor target features among the presented stimuli before trying them as potential target features).

The accuracy quickly raised after participants learned the target color and dropped back to chance level when participants were instructed that the target-defining feature changed at the end of the first block. Once participants learned that orientation defined the target in the second block, accuracy quickly rose again. However, the accuracy never reached the high levels of the first block, indicating that searching for a specific orientation was harder than searching for a specific color. As in Experiment 1, response validity effects were restricted to cues matching the current targets’ defining feature and occurred swiftly after the target-defining feature was learned.

#### Response validity effects after the target feature changed

To investigate whether the target feature previously used as the search criterion (color) keeps capturing attention after participants were instructed that the target is now defined by a different feature (at the start of the second block), we analyzed the last two bins of the color block and the first two bins of the second (orientation) block. All participants in this analysis knew the target feature in the color block and did not yet know the target feature in the orientation block. However, since orientation was not yet learned as the target feature, a matching or nonmatching orientation did not yet exist for the participants. Therefore, we reduced the four cue conditions to two based on whether the cue color did or did not match the previous target feature. This allowed us to measure a potential attentional capture by cues with the previous target color with a bin size of 32 trials while keeping the measurement reliability acceptable.

**Fig. 7:**
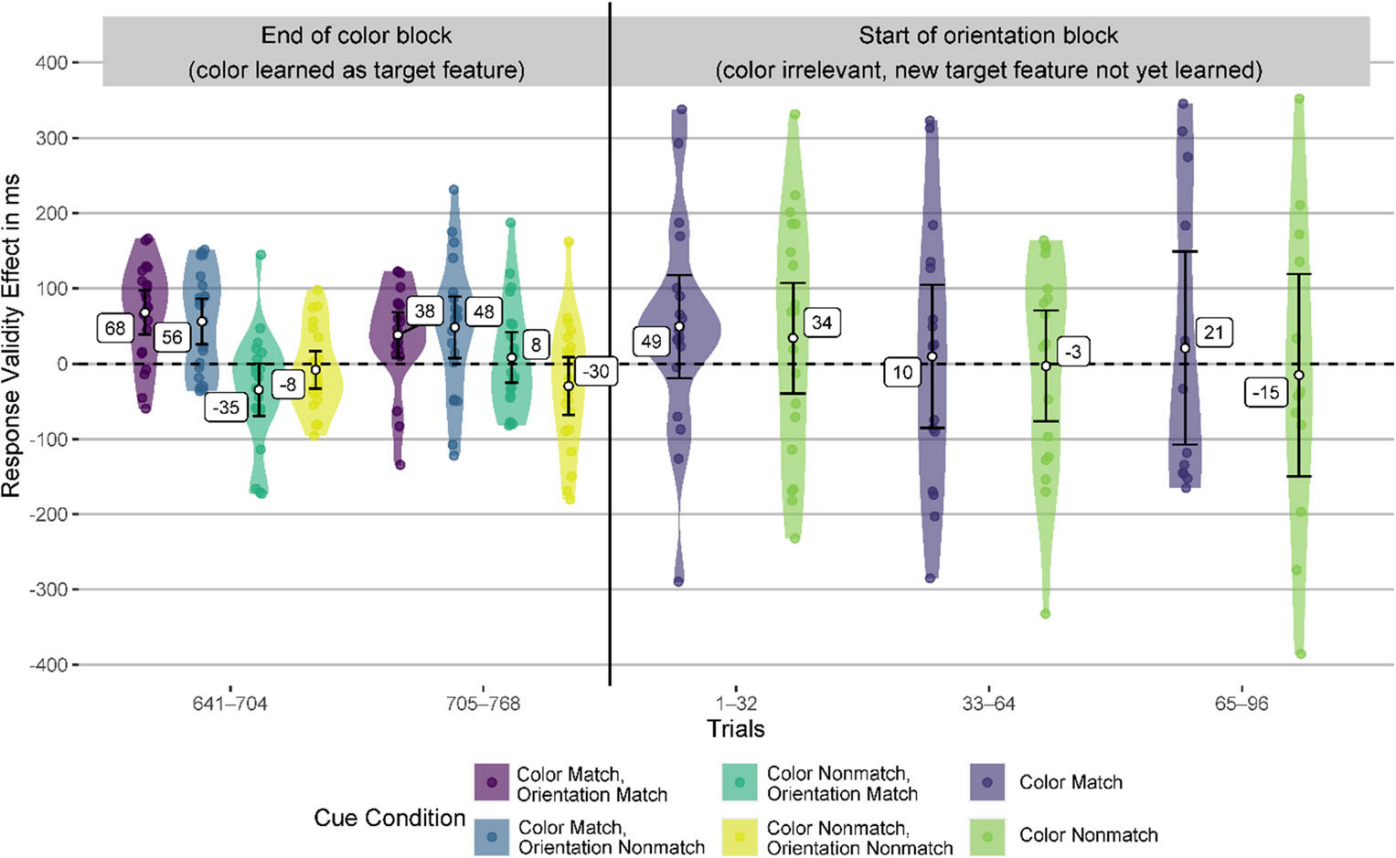
Validity effects in Experiment 2, before and after the target feature changed. This figure depicts the validity effects in the bins (64-trial or 32-trial sequences, depending on the block) before and after the target feature changed. The error bars represent the 95% CI from the one-sample *t* test against zero. (Color figure online)

In the last two bins of the color block, we found significant response validity effects for the cues with a matching color (38 to 68 ms, all *p*s < .023). However, the response validity effects for the cues with a nonmatching color were not significantly different from zero (−35 to 8 ms, all *p*s > .051). In the first 32-trial bin in the orientation block—where participants were instructed that color is not task relevant anymore—cues in the previous target color elicited a response validity effect of 49 ms. However, due to the high variance (*SD* = 142 ms), this effect is not significantly different from zero (*p* = .15, *d*_unb_ = 0.33). Cues in the nonmatching color elicited a similar response validity effect that was also not significantly different from zero due to high variance (34 ms, *SD* = 157, *p* = .35, *d*_unb_ = 0.21). After that initial bin, we found much smaller response validity effects for all cues (−15 to 21 ms, *d*_unb_ −0.07 to 0.05, all *p* values above .73). Figure 7 depicts the response validity effects, and Table 6 in the Appendix provides all statistical information and effect sizes.

### Discussion

We replicated Experiment 1, except that participants had to relearn the target-defining feature. In the first block, they learned that a specific color defined the target. At the beginning of the second block, we instructed them that color is no longer task relevant and that learning the new target-defining feature is necessary. This change allowed us to investigate a potential attentional bias toward previously selected features when participants have not yet acquired an attentional control setting that enables the search for the new target (from the beginning of the second block until they learned the new target-defining feature). If a potential influence of selection history was disguised because it was incompatible with the attentional control setting elicited by the instruction in Experiment 1, we should have found such influences as long as no attentional control setting is established.

The results after participants knew the target-defining features were the same as in Experiment 1: Cues captured attention only if they matched the current target-defining feature with no evidence for an attentional bias towards cues matching the previously learned and selected feature. However, during the first 32 trials of the orientation block (after participants were instructed that color is now target irrelevant), cues with the previous target color elicited a response validity effect similar to the color-matching cues in the color block. Interestingly, nonmatching color cues elicited similar response validity effects as well. Although these effects were not significantly different from zero, the response validity effects in the following bins were much smaller, indicating that there might be a true effect in the first bin that would need more power to find as significant. Increasing the bin size to 64 trials would increase the power, but the effect is so short-lived that it is vastly reduced when averaging across 64 trials.

If we assume that those response validity effects are not only statistical artifacts, it is interesting that not only the previous target color but also the nonmatching color captured attention for a short time after the target feature changed. This unspecific capture might indicate bottom-up capture by the salient cues independent of color, although it is unclear why this capture stopped after about 32 trials. One trivial explanation could be that (some) participants did not believe our instruction that color was task irrelevant and tried searching for color to see for themselves. After a few trials receiving negative feedback, they switched to a different search criterion, resulting in vanishing response validity effects for all cues from the second bin onwards. However, such a general search for color would be counterintuitive since participants knew that color is no longer a target-defining feature.

Either way, the results of Experiment 2 do not support the hypothesis that previously selected target features keep capturing visual attention automatically. Instead, our findings suggest that explicit knowledge of what is not a target feature is enough to suppress a potential lingering selection bias towards this nontarget feature. This result could also indicate that participants established a “negative” attentional control setting for the no longer relevant and, hence, to-be-suppressed previous target color, based on the knowledge of what the target is not (here, not defined by color)^3^, and this attentional control setting might have overwritten the selection bias similar to the “positive” attentional control setting in Experiment 1.

However, when awareness or explicit knowledge about what is and what is not task relevant (i.e., top-down influences) dominates attentional guidance, selection history influences of explicitly learned target features might only occur if they are compatible with equally awareness-dependent top-down control settings. This hypothesis is also plausible in real-world scenarios: If we search for the green jacket of a friend in a crowd, it would be counterproductive if this visual search were impaired because we previously looked for blue jackets in a shop. However, the shape of the previously looked-for jackets could benefit the later search for the green jacket since an attentional bias towards the shape of jackets would be compatible (or at least not incompatible) with the new search goal. Therefore, in Experiment 3, we tested whether learning of target features influences attention guidance when target features are compatible with top-down goals, and we also investigated their awareness-dependence—that is, whether explicitly known but unused target-defining features influence attentional guidance.

## Experiment 3

In Experiment 3, we used a procedure based on the redundant relevant “cue” (RRC) protocol (cf. Trabasso & Bower, 1968), where two redundant and relevant “cues,” here, features, predict an outcome (in our case: successful search). For example, in past studies, participants had to classify stimuli consisting of different shapes in different colors, where green circles belong to Class A and red squares to Class B. Other shapes and colors were irrelevant, but all circles were green, and all squares were red. Therefore, these features were redundant and both relevant. In Experiment 3, participants had to learn the target-defining feature as in the previous experiments. However, now the target was defined by a unique color (red) and orientation (horizontal). Both features are redundant since they could both be used to search for the target.

Earlier studies showed that usually only one of the redundant features is learned as target defining (e.g., Bourne Jr. & Haygood, 1959; Hara & Warren, 1961; Warren & Warren, 1969). However, with overtraining, the second redundant feature often is learned as target defining as well (e.g., Sutherland & Holgate, 1966; Trabasso & Bower, 1968). Overtraining means that the discrimination task is continued even after one target-defining feature has been learned, which is the case in our Experiment 3. Therefore, we assumed that at least a proportion of our participants learned both redundant features as target defining.

However, based on the performance difference (overall accuracy) between color blocks and orientation blocks in our Experiments 1 and 2, we further assumed that participants would nevertheless preferentially use the target-defining color to search for the target (e.g., due to a lower salience of the target-defining orientation than of the target-defining color). Remember that the accuracy in the second (orientation) blocks of Experiments 1 and 2 never reached that in the first (color) blocks. Therefore, participants had to report whether they noticed that the color and orientation both defined the target and which feature they (mainly) used to search for the target (or whether they used both) at the end of the present experiment. Thus, we can clarify the role of the participant’s explicit knowledge or their awareness of the target-defining features for attentional guidance. For example, features might only guide attention if participants know (and are aware) that they define the target. This would also explain why instructions superseded the influence of learning and selection history in the preceding experiments, as the instructions and learning processes both resulted in explicit knowledge about the target-defining feature. Moreover, explicit knowledge can be updated easily—unlike relatively long-lasting (automatic) responses to stimuli after learning processes.

In any case, by using redundantly defined targets, it is possible that participants learned both target-defining features but only used one to search for the targets. For example, participants might have used only color to search for the target, simply because color is somewhat easier to search for than orientation. This might happen despite our participants’ acquired knowledge regarding both target-defining features.

If participants would mainly use color to identify the target, we could simultaneously investigate the influence of the used search criterion and a consistently selected/processed^4^ but maybe not voluntarily used target feature (a selection history influence). Moreover, both these influences are compatible and would benefit visual search performance. To test these influences on attentional guidance, we used four different cues consisting of both (redundant) target features (hereafter referred to as full-matching cue), with only one of the target features (color-matching or orientation-matching cue), or without any target feature (nonmatching cue). We expected that cues matching the search criterion used during visual search guided attention. It is open, however, if unused or unnoticed target-defining features that were repeatedly selected influence attentional guidance when compatible with the search goal (cf. Foerster & Schneider, 2018)—as in the present experiment. Nonmatching cues should never guide attention, while full-matching cues might have an advantage over cues matching only one target feature if both target-defining features have an additive effect on attentional guidance.

### Method

#### Participants

Twenty-three participants (19 women, four men) aged between 18 and 30 years (*M* = 21.43, *SD* = 4.03, *Mdn* = 20) took part in Experiment 3. One participant was excluded due to a high error rate even after learning the target-defining feature (21.17%, an outlier according to a one-sided Grubbs test, *p* = .019). The participants were tested under the same conditions as in the previous experiments.

#### Design and procedure

As in the previous experiments, participants had to learn to identify the target by trial and error. Since the color and orientation of the target were unique, only one of these features was needed to search for the target successfully. The learning phase ended after the participants reached at least 80% accuracy during the last 20 trials. Next, they typed the discovered rule into a dialog box and proceeded with the experiment after a self-paced break. After that, the testing block started, consisting of 1,280 trials (80 valid and 240 invalid trials per cue condition; see below). To avoid any possible influence of the cues on learning the target-defining feature(s), we did not present any cues during the initial learning phase. At the end of the experiment, participants had to report whether they noticed that the target was defined by its unique color and orientation and which feature they mainly used to search for the target via typing the answers into dialog boxes. Additionally, the participants were asked about their search strategy and whether they used both target-defining features (in case they noticed both features as target defining).

The sequence of the trials was similar to the previous experiments. However, the target display was shown for 300 ms instead of 400 ms. The placeholders were slightly thinner (0.08°), and the gaps narrower (1.13°). The stimuli were presented at the same positions as in the previous experiments. The cueing display (only presented after learning) consisted of four lines. One of these lines was the singleton cue, which was either fully matching the target (a red horizontal line), matching only the target color (a red line, tilted 45° to the left), matching only the target orientation (a cyan horizontal line), or nonmatching (a cyan line, tilted 45° to the left). The other three lines in the cueing displays were all nonsingletons, either all vertically oriented or all tilted 45° to the right, and either all green or all yellow if the cue was red, and either all magenta or all yellow if the cue was cyan.

**Fig. 8:**
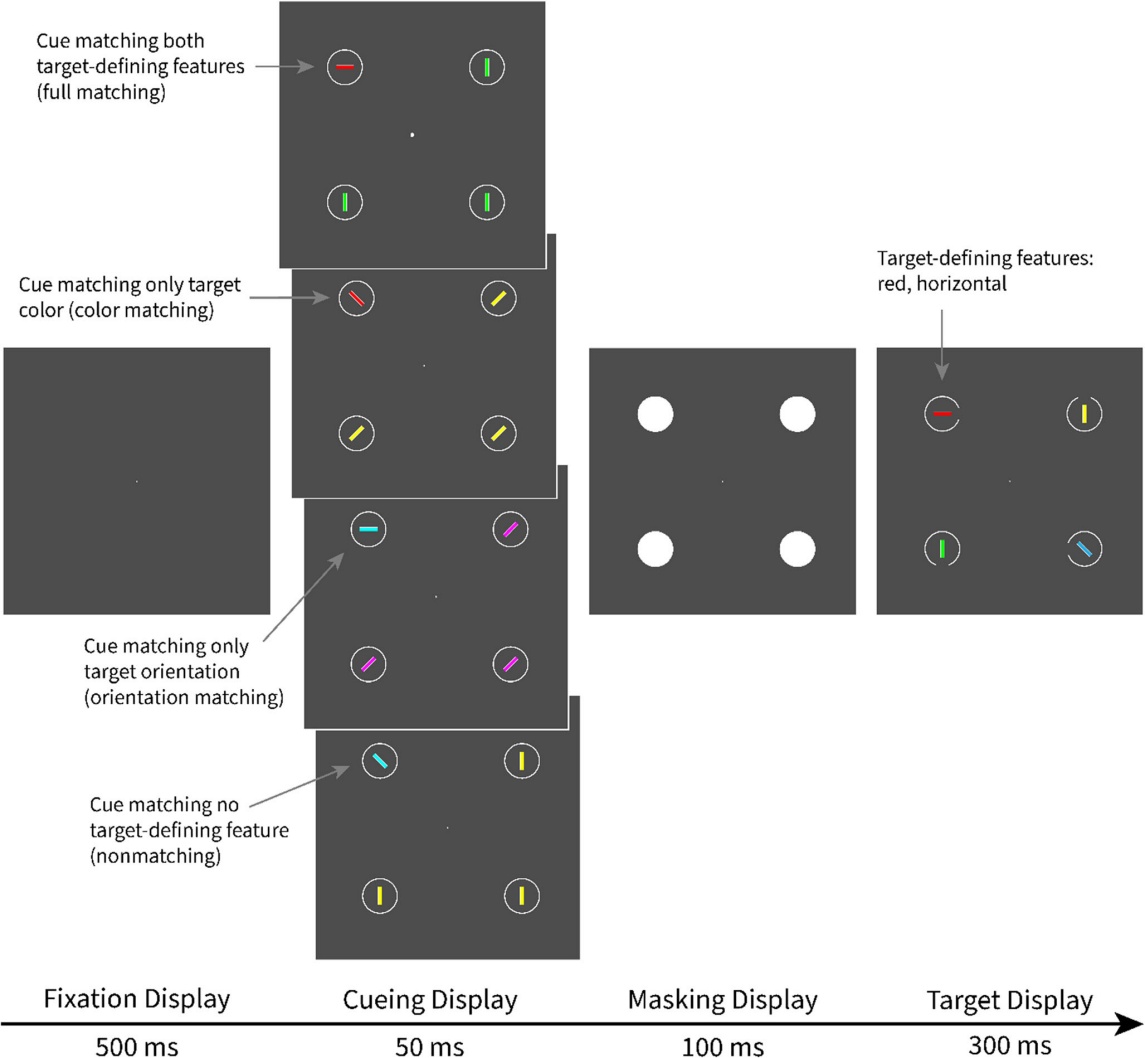
Procedure of Experiment 3. This figure depicts a valid trial with all four cueing conditions. During learning, the circles in the cueing display were empty. Otherwise, the procedure was the same. Not depicted is the response display (shown for 1 s or until response) and the feedback display (shown for 1 s). Both followed the target display. The stimuli are drawn to scale, but the gray background is cropped and does not represent the screen size. (Color figure online)

After the cueing display (only presented after learning), a masking display was presented for 100 ms, consisting of four white disks, each covering one circle and line in the cueing display. Then the target display appeared for 300 ms, consisting of a red horizontal line (the target) and three distractors (a green, a blue, and a yellow line), one per each of the same positions as were used for the stimuli in the cueing and masking displays. Two of the distractor lines in the target display were vertically oriented, and one was 45° tilted to the left, or vice versa. See Fig. 8 for depictions of cueing, masking, and target displays.

In a variant of the experiment, the target was the green vertical line. Accordingly, in the cueing display, the roles of the colors red and green were interchanged, as well as the roles of vertical and horizontal lines. Otherwise, the procedure was the same. Participants were randomly assigned to one variant of the experiment.

### Results

Other than testing how fast participants learned and ensuring that the learning criterion of 80% correct performance was met, we did not analyze data in the learning block since no cues were presented in this block. In the testing block, we excluded 7.41% incorrectly answered trials from all validity effect analyses. After the exclusions 75 (*SD* = 3) valid trials across all cue conditions remained on average. The average ICC1 within cue conditions was .14, and the ICC2 was .95. For the entire dataset, the ICC1 was .13, and the ICC2 was .99.

#### Learning curve

**Fig. 9:**
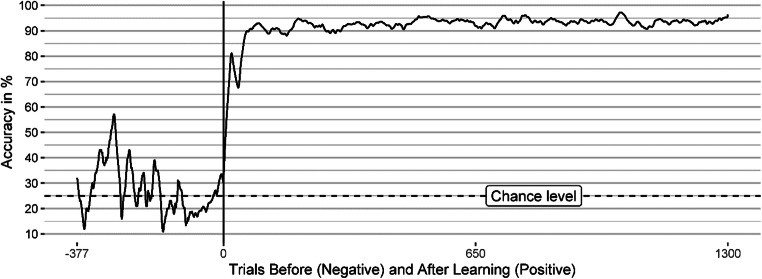
Learning curve in Experiment 3. This figure depicts the accuracy over time, smoothed using a moving average of five trials. The *x*-axis represents the trial sequence centered around the trial in which participants learned at least one target-defining feature (marked with a vertical line). Negative trial numbers indicate trials before and positive trials after learning

We analyzed the learning curve using the same method as in the previous experiments, although without the validity effect since we did not present cues before participants learned to identify the target. All participants learned to identify the target within a maximum of 397 trials (*Mdn* = 20). Furthermore, all participants reported that they learned and used the target color to search for the target. Only 13 participants reported noticing that the target was also defined by its unique orientation. However, they kept using the target color to find the target (see also below). The learning process was also similar to the previous experiments. After the participants learned to identify the target, the accuracy rate quickly approached the highest accuracy (see Fig. 9).

#### Validity effect

**Fig. 10:**
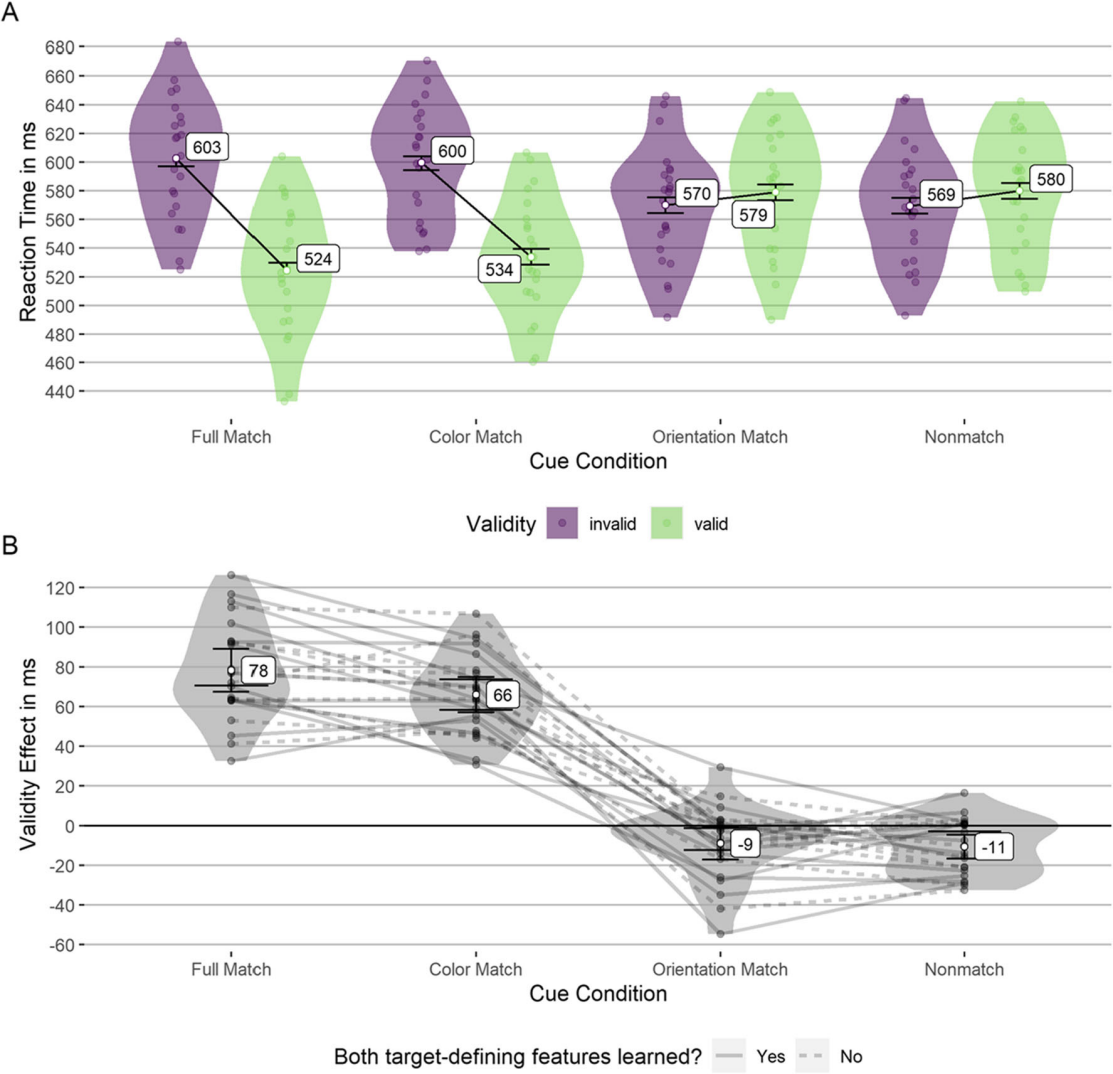
Mean validity effects following learning in Experiment 3. This figure depicts the reaction times (**A**) and validity effects (**B**) for all cue conditions in Experiment 3. The values per participant are plotted to show their distribution. Values of the same participant are connected with lines (only **B**) to give an impression of the measurement reliability. The narrow error bars represent the 95% CI for the one-sample *t* test against zero. The wide error bars represent the 95% CI for the difference between the cue conditions (only **B**). The most extreme values have only one error bar since these values can only be compared with a less extreme value. No overlapping indicates a significant difference. The factor “both target-identifying features learned” indicates whether the participants noticed that the target was defined by two features simultaneously (see Table 4 for the results separated by this factor)

**Fig. 11:**
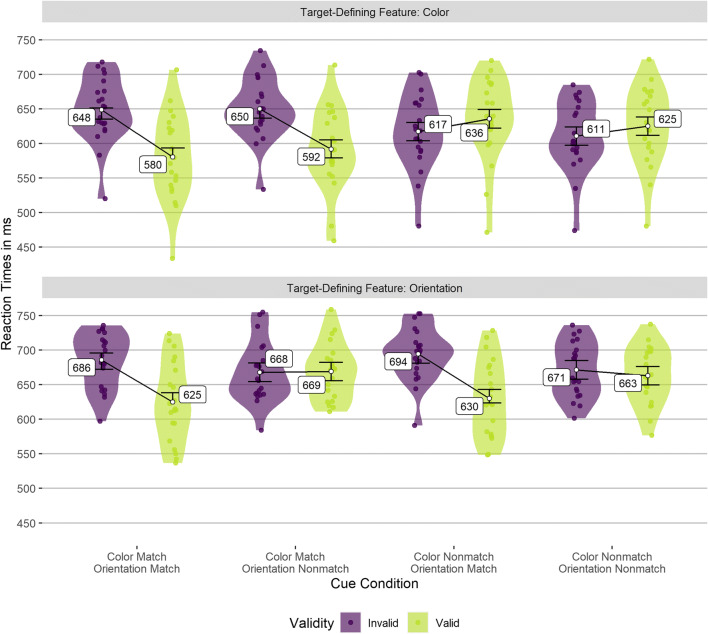
Reaction times (following learning in color block) in Experiment 1. This figure depicts the reaction times for all cue conditions in each block. The error bars represent the 95% CI for the difference between the conditions in each block. No overlapping indicates a significant difference

**Fig. 12:**
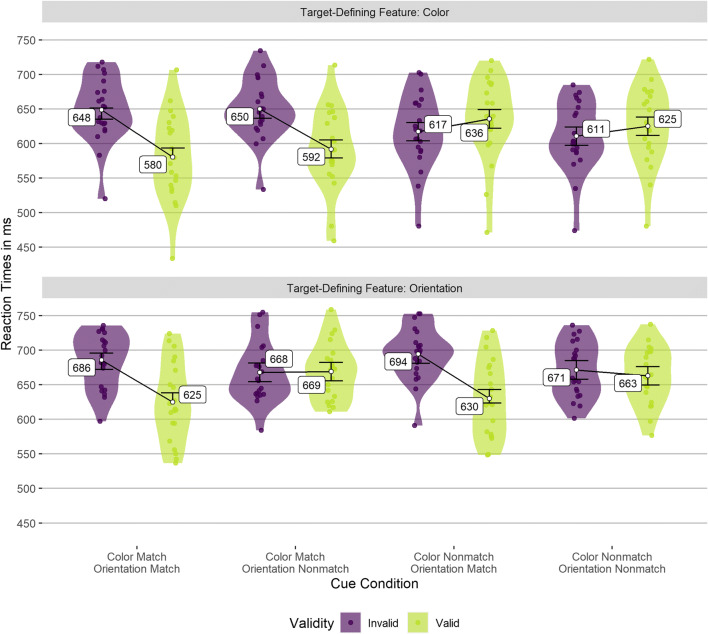
Reaction times (following learning in both blocks) in Experiment 2. This figure depicts the reaction times for all cue conditions in each block. The error bars represent the 95% CI for the difference between the conditions in each block. No overlapping indicates a significant difference

The validity effects in the testing block (after learning) were normally distributed in all conditions (tested with Shapiro–Wilk tests, all *p* values above .193). We found significant positive validity effects for the full and color-matching cues, a small but significant negative validity effect for the nonmatching cue, and no significant validity effect for the orientation-matching cue (see Table 3). The difference between full- and color-matching cues was not significant, as was the difference between orientation-matching and nonmatching cues (see Fig. 10). According to simulations, the achieved power was 96.1% to find a validity effect of 20 ms as significant above zero (23 participants, *α* = .05).

**Table 3:** Mean validity effects following learning in Experiment 3

Cue condition	*M* (*SD*)	95% CI	*t*(*df*)	*p* ^a^	*d*_*unb*_ ^b^	95% CI
Full match	78 (25)	[68, 89]	15.08(22)	<.001	3.04	[2.13, 4.15]
Color match	66 (20)	[57, 75]	15.53(22)	<.001	3.13	[2.2, 4.26]
Orientation match	−9 (19)	[−17, −1]	−2.28(22)	.068	−0.46	[−0.9, −0.04]
Nonmatch	−11 (14)	[−17, −5]	−3.63(22)	.004	−0.73	[−1.22, −0.28]

Results from two-sided one-sample *t* tests against 0. Mean and *SD* in ms

^a^Corrected for multiple comparisons using the method of Benjamini and Yekutieli (2001)

^b^Effect size standardized using the group *SD* and corrected using Hedges’s correction factor (Hedges, 1981)

Additionally, we analyzed the validity effects separately for participants who noticed that the target always had the same color and orientation (*n* = 13) and those who did not (*n* = 10). The results are presented in Table 4. Participants who noticed the redundancy (which indicates learning and resulting explicit knowledge of both target-defining features) showed a significantly stronger validity effect (Δ 18 ms) in the full-matching cue condition than in the color-matching condition (where the line orientation was nonmatching). Therefore, the (additionally) matching orientation of the singleton cue in the full-matching cue condition had an attention-guiding or an attention-keeping effect. However, the difference between the orientation-matching cue and the nonmatching cue was not significant, indicating that this attentional effect only occurred if the cue matched a “main search criterion” (here, for target color). If the participants did not notice the target’s feature redundancy, there was neither a significant validity effect difference between the full- and color-matching cues nor between the orientation-matching and nonmatching cues. Simulations showed that this analysis achieved a power of 63.1% to find a 25 ms higher validity effect in the full-match condition than in the color-match as statistically significant. For a 30 ms difference, the achieved power was 80.4% (10 participants, *α* = .05).

**Table 4:** Mean validity effect contrasts following learning in Experiment 3

Contrast	*M* (*SD*)	95% CI	*t*(*df*)	*p* ^a^	*d*_*unb*_ ^**b**^	95% CI
Both target features noticed						
Full–Color	18 (20)	[6, 30]	3.17(12)	.033	0.82	[0.22, 1.51]
Full–Orientation	93 (25)	[78, 107]	13.58(12)	<.001	3.53	[2.18, 5.34]
Full–Non	90 (25)	[75, 106]	12.85(12)	<.001	3.34	[2.05, 5.06]
Color–Orientation	75 (26)	[59, 90]	10.53(12)	<.001	2.73	[1.63, 4.18]
Color–Non	72 (18)	[61, 83]	14.4(12)	<.001	3.74	[2.32, 5.65]
Orientation–Non	−2 (18)	[−13, 9]	−0.45(12)	1.00	−0.12	[−0.67, 0.42]
Only color as target feature noticed						
Full–Color	5 (14)	[−5, 15]	1.11(9)	1.00	0.32	[−0.3, 0.98]
Full–Orientation	80 (22)	[65, 96]	11.5(9)	<.001	3.33	[1.87, 5.39]
Full–Non	87 (23)	[70, 104]	11.87(9)	<.001	3.43	[1.93, 5.56]
Color–Orientation	75 (25)	[58, 93]	9.53(9)	<.001	2.76	[1.5, 4.5]
Color–Non	82 (24)	[65, 100]	10.72(9)	<.001	3.1	[1.72, 5.04]
Orientation–Non	7 (12)	[−2, 15]	1.78(9)	.41	0.51	[−0.12, 1.22]

Mean and *SD* in ms. Full = full match; Color = color match; Orientation = orientation match; Non = nonmatch cue condition

^a^Corrected for multiple comparisons using the method of Benjamini and Yekutieli (2001)

^b^Effect size standardized with the *SD* of the means or per-participant differences, respectively, and corrected using Hedges’s correction factor (Hedges, 1981)

### Discussion

The learning curve in Experiment 3 was similar to that in the first two experiments, indicating sudden learning through insight (cf. Spence, 1940): After participants learned to identify the target, the accuracy rate increased quickly from chance level to the maximal accuracy rate and remained there until the end of the experiment. All participants reported using the target color to search for the target. Only 13 participants reported noticing that the target was also identified by its unique orientation. Both results are consistent with the results of Experiments 1 and 2 (lower accuracy in orientation blocks compared with color blocks) and with earlier research showing that salient features are easier to associate with the target and, therefore, learned faster (cf. Kamin & Schaub, 1963; Trabasso & Bower, 1968; Werchan & Amso, 2020). Nevertheless, the other (redundant) target-defining feature is sometimes learned and explicitly registered as well over time (i.e., with overtraining) by a considerable proportion of participants (human or nonhuman; cf. Suchman & Trabasso, 1966; Sutherland & Holgate, 1966).

The selective use of color to search for the target (as reported by the participants) was reflected in attentional guidance: Cues similar to the target color strongly guided attention, whereas cues similar to the target orientation only elicited a small negative validity effect similar to nonmatching cues. Although the small negative validity effects are statistically significant, we consider them too minuscule to warrant interpretation. Importantly, these cues, thus, seemingly did not guide attention, despite their target association and, hence, prior history of consistent selection (together with all other target features).

In addition, full-matching cues elicited significantly stronger validity effects than color-matching cues if participants reported noticing both target-defining features (color and orientation). If participants did not report noticing both target-defining features, the full-matching cues had no advantage over the color-matching cue. This result suggests that the small selection history influence of the target-defining feature otherwise unused as search criterion was contingent on the cues matching the self-reported color-search criterion, since orientation-matching cues in a nontarget color did not show any evidence of attentional guidance (however, there might be a small effect we did not find due to insufficient power). It seems that the self-reportedly noticed and used search criterion is the primary influence on attentional guidance, and possible influences of selection history only occurred if they are compatible with that search criterion and only if participants were aware of the unused features as target-defining.

The relatively small effect advantage of full-matching versus color-matching cues might be due to some participants noticing the orientation as a target-defining feature only after they were asked for it at the end of the experiment. Others might have genuinely noticed both target-defining features but the orientation only at a later stage of the experiment. In each case, the influence of selection history on attentional guidance would be attenuated.

However, an implicit influence of selection history based on repeatedly selecting target-defining features (color and orientation) should be independent of explicitly noticing the task relevance of a repeated feature (e.g., Kristjánsson & Driver, 2008). Although the unused target-defining feature (orientation) was compatible with the search goal and using it would even benefit target identification, we did not find any validity effects of cues similar to the target orientation. Furthermore, the absence of any instruction to focus on only one target feature is particularly inviting for selection history influences since there is no reason for participants to suppress attentional guidance toward cues matching the target orientation. Having said this, it is still possible that participants did not encode and implicitly learn the unused target feature. However, this is far from trivial, as selection history has sometimes been considered a form of bottom-up or implicit process (cf. Theeuwes, 2013). As incidental learning is arguably a hallmark of implicit processing (cf. Perruchet & Pacton, 2006), our results are at variance with this radical interpretation of selection history effects since they suggest that explicit knowledge is a prerequisite of selection history effects.

In conclusion, corroborating our previous results, we found no evidence for an influence of selection history on attentional guidance independent of top-down guidance. We only found a small effect of selection history for cues matching a used search criterion. However, there might be a small effect of selection history which we did not find due to insufficient statistical power. Thus, we can only conclude that such an effect would likely be smaller than 30 ms since we would have found such an effect with a power above 80%.

## General discussion

In a recent review, Luck et al. (2021) described “the extent to which explicit goals and/or selection history can exert proactive control over the gain of nonspatial features prior to saliency computations” and “whether explicit goals and implicit learning operate independently or are integrated into a unitary control state,” as two major points of disagreement between bottom-up and top-down views of visual attention (p. 4). Our current study provides new evidence on both questions and adds to a recent surge of studies reevaluating the influence of selection history on attentional guidance (e.g., Jiang et al., 2015; Luque et al., 2021; Ramgir & Lamy, 2021).

In Experiment 1, we investigated whether there is a lingering influence on attentional guidance from previously learned target features once a new target feature is instructed. In contrast to claims that selection history influences attentional priority persistently and even if no longer relevant, we found no evidence that the previous target feature captured attention if that feature is no longer task relevant. That is, a feature learned, used, and selected in each trial did not guide visual attention during a subsequent instructed search for a different target feature. One reason for the lack of influence of selection history might be that the instruction to search for the new feature immediately induced attentional control settings for the new target feature. However, the new target feature was incompatible with the previously learned feature and might have overridden the possible lingering bias of selection history.

To test whether selection history biases attentional guidance in instruction-induced attentional control settings, in Experiment 2, after learning to search for one target-defining feature, participants were instructed that the previous target feature is no longer task relevant and to learn the new target-defining feature. After the participants learned the new target feature, we found similar results as in Experiment 1. However, after the end of the first (color) block—when we instructed participants that the previous target feature was no longer relevant—all cues tended to capture attention in the first 32-trial bin. Although the unspecific capture of all cues might be related to the previous use of color as the search criterion, it is very short-lived and not selective to the previous target feature. Thus, whether these response validity effects represent the automatic capture of previously selected target features remains unclear.

To summarize the first two experiments, we did not find clear evidence of a lasting influence of a previously searched-for and selected target-defining feature on attentional guidance during search for a new target feature (Experiment 1) or after participants learned that the previous target feature is no longer relevant (Experiment 2). The results of Experiment 2 indicate that the specific instruction-induced attentional control settings did not disguise a potential influence of selection history in Experiment 1. It seems that instructions and explicit knowledge about task relevance exert a much stronger influence on attentional guidance than selection history.

A possible explanation for this dominance is that the previous target feature was always at odds with the new information about task relevance. In Experiment 3, we tested the possibility that selection history effects must be compatible with current search goals to influence attentional guidance. While instructed features and learned features were uncorrelated in Experiments 1 and 2, we used two redundantly target-defining features in Experiment 3. In addition, participants were allowed to learn to use both of these features. Thus, in Experiment 3, whichever of the two target-defining features participants used to search for the targets, the possible influence of selection history of the alternative target-defining feature was compatible with the used search criterion. However, while two features (color and orientation) defined the target (e.g., a red horizontal line), it turned out that participants used only color to search for the target. Although not used as a search criterion, the target orientation was also selected in each trial throughout the experiment. Nevertheless, only 13 out of the 23 participants reported noticing that two features defined the target at the end of the experiment. Critically, only those participants that were aware of the orientations as target-defining features showed some evidence of attentional guidance by these features. Jointly, the results from all experiments suggest that instructions can dominate selection history in attentional guidance to the extent that these selection-history effects themselves depend on the participants’ explicit knowledge and deliberate use of the learned target features. This dependence on the explicit usage of learned target features allows top-down influences based on instructions to replace selection-history influences.

In summary, the results indicated that the used search criterion is the predominant influence on attentional guidance and that the learned criteria were used much as an instruction to set up a search criterion for attentional guidance. In contrast, in Experiment 3, a consistently selected but unused target orientation did not capture attention; only cues with a feature that matched the participants’ deliberately chosen search criterion did. That is, cues matching only the target orientation did not elicit significant validity effects, and cues matching both target features did not elicit stronger validity effects than cues matching only the target color unless participants noticed both target-defining features. In this case, they showed slightly stronger validity effects for full-matching cues than cues matching only the target color. Except for this awareness-dependent influence of selection history, there was no evidence that selection history exerted a measurable influence on attentional guidance independent of top-down influences in our experiments, which indicates that selection history does not influence attentional guidance but other processes that facilitate visual search.

### Previous results and limitations

Our findings seemingly contradict previous studies arguing for a lingering selection bias based on previous attentional deployments (cf. Awh et al., 2012). However, our results offer an extension or clarification rather than a refutation of selection history effects. We investigated specifically attentional guidance using a spatial cueing protocol with multiple cue conditions, which allows measuring spatial attentional allocation without confounding influences of response processes and target–distractor interactions in the target display. Although this design was used previously to investigate priming effects (cf. Belopolsky et al., 2010; Schoeberl et al., 2019), we focused on a potential lingering selection bias towards previously learned target-defining features used as the search criterion to accomplish an earlier task. Since the to-be-learned feature was necessary for the task, the learning was arguably explicit. Only in Experiment 3, participants who failed to notice the redundant target feature might have learned the unnoticed feature implicitly. However, whether they did remains speculative since we found no selection bias indicative of previous implicit learning. This finding implies that participants either failed to use or even acquire any memory of a target-defining feature once another such feature was registered and successfully used. As incidental learning is arguably a hallmark of implicit processing (cf. Perruchet & Pacton, 2006), this result contradicts the most radical bottom-up theories of selection history effects on attention guidance (cf. Theeuwes, 2013).

Nevertheless, using instructed or learned target features has been shown to bias subsequent search performance (Feldmann-Wüstefeld et al., 2015; Kadel et al., 2017) but not always (Anderson & Halpern, 2017, Experiments 2a and 2b). In these studies, selection history was measured with the distraction caused by the previous target feature when presented simultaneously with a new, different target in a subsequent task. For example, participants searched for a red or green target (Anderson & Halpern, 2017) or learned that color is the response-relevant feature (Feldmann-Wüstefeld et al., 2015; Kadel et al., 2017). In a subsequent new task, participants have to search for a shape singleton (e.g., a diamond among circles) and respond to the orientation of a line inside the target shape. In the studies of Feldmann-Wüstefeld et al. (2015) and Kadel et al. (2017), in half of the trials, one of the nontarget shapes was colored red (distractor present condition). In the other trials, all shapes were gray (distractor absent condition). Results showed that the presence of the distractor slowed reaction times compared with distractor absence trials, and this slowing was more pronounced in participants who previously learned that color was response-relevant than in participants who learned that shape was response relevant. Notably, participants who learned the relevance of shape are usually distracted by the additional color singleton as well (Feldmann-Wüstefeld et al., 2015; Kadel et al., 2017; see also Theeuwes, 1992). One explanation why the distractor captures attention is because a singleton distractor is task-relevant during a singleton search (cf. Ansorge et al., 2010; Bacon & Egeth, 1994). The selection history effect found by Feldmann-Wüstefeld et al. (2015) and Kadel et al. (2017) consisted of a stronger capture by a distractor that additionally matched the previously learned response-relevant feature, which is very similar to our conclusion of Experiment 3 (selection history influence is contingent on top-down search goals).

In contrast, Anderson and Halpern (2017) presented the previous target color distractor among other (neutral) colors, making the distractor a nonsingleton. Thus, the distractor does not match the search criterion for singletons. When the previous target color was associated with high reward, Anderson and Halpern (2017) found significant distraction by that color during a subsequent search for a shape singleton. However, if the previous target color was not rewarded but only selected, no distraction was found, indicating no attentional capture by the previous target color (Experiments 2a and 2b)—which is also consistent with our results.

Another line of research found decreased search performance if a previous target letter appeared together with a new target letter compared with trials without a previous target letter (Kyllingsbæk et al., 2001; Shiffrin & Schneider, 1977). In the experiment most similar to our experiments (Kyllingsbæk et al., 2001; Experiment 4), five participants had to respond to the presence or absence of an instructed target letter among a consistent set of five distractors over eight sessions with 1,000 trials over four days. After that, during two testing sessions, one distractor letter was redefined as the new target, and the previous target letter became one of the possible distractors. Otherwise, the set of distractors remained the same. Each of the distractors (including the previous target letter) was presented equally often.

Search performance (measured with *d′*, an indicator of discriminability, here, between target-absent and target-present trials) was decreased if the previous target letter occurred as a distractor, but the effect was very small and often not significant but arguably reliable since it was found in all five participants (Kyllingsbæk et al., 2001; Experiment 4). However, the results could be influenced by learned distractor suppression. In the testing sessions, when there was no previous target present (previous target-absent trials), all distractors were from the same set as in the learning sessions. In previous target-present trials, one of the distractors was the previous target. With the small set size of only three stimuli, target-present trials without a previous target letter consisted of double the number of well-known distractors than target-present trials with a previous target letter as a distractor (2 vs. 1). In target-absent trials, this relation was three to two). Since consistent distractor features have been shown to elicit suppression (cf. Ruthruff et al., 2021)—and such suppression would be beneficial for performance in a target detection task—it could be that the better discriminability was caused by more efficient distractor rejection in trials without a previous target letter as a distractor. Unfortunately, Kyllingsbæk et al. (2001) did not report how the presence of a previous target letter influenced the hit rate of target-present trials compared with target-absent trials. Nevertheless, this example shows how selection history can influence search performance without necessarily influencing attentional guidance.

While we consistently failed to find a long-lasting attentional bias towards previously learned and selected features, our conclusions about potential short-lived biases are limited by low statistical power. The validity effect analysis over time suffered from the trade-off between high temporal resolution and measurement reliability. Therefore, we cannot exclude that the previous target color captured attention during the first few trials of the new task. Further research on the influence of target feature awareness is warranted since the power in our third experiment was undesirably low for some analyses.

We conclude that selection history can sometimes be prematurely accepted as a third influence on attentional guidance independent of bottom-up and top-down influences and that alternative explanations are sometimes not sufficiently considered. For example, previous experience with a specific target feature might improve that feature's processing and recognition only after attention is allocated based on attentional priority or the subsequent response selection (cf. Hillstrom, 2000; Ramgir & Lamy, 2021). Such influences improve visual search without necessarily affecting attentional guidance.

Furthermore, our results suggest that an instructed or used search criterion exerts a dominant influence on attentional guidance, whereas selection-history effects themselves depend on explicit knowledge. In this situation, selection history influences incompatible with the currently instructed search goals do not bias attentional guidance. However, maybe (partly) compatible and explicitly known but unused target features influence attentional guidance, showing an interesting interaction between top-down influences and selection history.

## Data Availability

The data of all experiments are available at Open Science Framework (OSF) and (10.17605/OSF.IO/P7W8C). None of the experiments reported here was preregistered.

## Appendix

### Reaction time plots

Appendix Figs. 11 and 12.

### Additional results

Appendix Tables 5 and 6

**Table 5:** Validity effects in Experiment 1, before and after the target feature changed

Cue	Trials	*M* (*SD*)	95% CI	*t*(*df*)	*p*	*d*_*unb*_^a^	95% CI
Color Block							
C & O +	641–704	52 (63)	[23, 80]	3.73(20)	.001	0.78	[0.31, 1.3]
C +, O −		47 (76)	[13, 82]	2.87(20)	.009	0.60	[0.15, 1.09]
C −, O +		−25 (62)	[−53, 3]	−1.87(20)	.076	−0.39	[−0.85, 0.04]
C & O −		−16 (46)	[−37, 5]	−1.56(20)	.13	−0.33	[−0.78, 0.1]
C & O +	705–768	70 (86)	[31, 109]	3.72(20)	.001	0.78	[0.31, 1.3]
C +, O −		57 (83)	[19, 94]	3.16(20)	.005	0.66	[0.2, 1.16]
C −, O +		−22 (59)	[−49, 5]	−1.73(20)	.099	−0.36	[−0.82, 0.07]
C & O −		6 (60)	[−22, 33]	0.44(20)	.66	0.09	[−0.33, 0.52]
Orientation Block							
C & O +	1–64	36 (91)	[−5, 78]	1.84(20)	.081	0.39	[−0.05, 0.84]
C +, O −		0 (89)	[−41, 40]	−0.02(20)	.98	−0.01	[−0.43, 0.42]
C −, O +		60 (93)	[17, 102]	2.95(20)	.008	0.62	[0.17, 1.11]
C & O −		3 (125)	[−54, 60]	0.11(20)	.91	0.02	[−0.4, 0.45]
C & O +	65–128	37 (65)	[8, 67]	2.65(20)	.015	0.56	[0.11, 1.04]
C +, O −		14 (93)	[−28, 57]	0.71(20)	.49	0.15	[−0.28, 0.58]
C −, O +		63 (91)	[22, 105]	3.19(20)	.005	0.67	[0.21, 1.17]
C & O −		−15 (88)	[−55, 25]	−0.8(20)	.43	−0.17	[−0.6, 0.26]

Mean and *SD* in ms. “C” = color; “O” = orientation; “−” = nonmatch; “+” = match

^a^Effect size standardized using the group *SD* and corrected using Hedges’s correction factor (Hedges, 1981)

**Table 6:** Validity effects in Experiment 2, before and after the target feature changed

Cue	Trials	*M* (*SD*)	95% CI	*t*(*df*)	*p*	*d*_*unb*_^a^	95% CI
Color Block							
C & O +	641–704	68 (64)	[39, 97]	4.85(20)	<.001	1.02	[0.51, 1.59]
C +, O −		56 (67)	[25, 87]	3.82(20)	.001	0.8	[0.33, 1.33]
C −, O +		−35 (76)	[−69, 0]	−2.08(20)	.051	−0.44	[−0.9, 0]
C & O −		−8 (55)	[−33, 17]	−0.67(20)	.51	−0.14	[−0.57, 0.29]
C & O +	705–768	38 (67)	[8, 68]	2.62(20)	.016	0.55	[0.1, 1.03]
C +, O −		48 (90)	[7, 89]	2.46(20)	.023	0.52	[0.07, 0.99]
C −, O +		8 (74)	[−25, 42]	0.51(20)	.62	0.11	[−0.32, 0.54]
C & O −		−30 (84)	[−68, 9]	−1.62(20)	.12	−0.34	[−0.79, 0.09]
Orientation Block							
C +	1–32	49 (142)	[−19, 118]	1.51(18)	.15	0.33	[−0.12, 0.81]
C −		34 (157)	[−39, 107]	0.97(19)	.35	0.21	[−0.23, 0.66]
C +	33–64	10 (179)	[−86, 105]	0.22(15)	.83	0.05	[−0.44, 0.54]
C −		−3 (144)	[−77, 71]	−0.09(16)	.93	−0.02	[−0.5, 0.45]
C +	65–96	21 (201)	[−107, 149]	0.36(11)	.73	0.1	[−0.47, 0.67]
C −		−15 (211)	[−149, 119]	−0.25(11)	.81	−0.07	[−0.64, 0.5]

Mean and *SD* in ms. “C” = color; “O” = orientation; “−” = nonmatch; “+” = match

In the color block, the target was defined by color and in the orientation block by orientation. For this analysis, we included only data from participants who knew the target feature in the color block and did not in the orientation block

^a^Effect size standardized using the group *SD* and corrected using Hedges’s correction factor (Hedges, 1981)

## Footnotes

1 Note that 75% was not the learning criterion used for analyses. In our analyses, we assumed successful learning if 80% correct responses were given in the last 20 trials.

2 CIs are generally not suitable for comparisons since they can be misleading. However, we used the comparison arrows from the R package *emmeans* to plot the error bars, which are designed to show a significant difference adjusted for multiple comparisons.

3 In case the negative target information has to be used to search for the target, Forstinger et al. (2022) found negative validity effects for the nontarget feature.

4 The target color was presented as a horizontal line. Thus, visual processing of the cue also involved processing its orientation. Nevertheless, color and orientation could still be used independently for attentional control settings and are not necessarily learned together.
